# Supplementary material for: Group psychosocial interventions for anxiety, depression, and post-traumatic stress disorder in children and adolescents in low- and middle-income countries: A realist systematic review and meta-analysis of randomised controlled trials
Source: PLOS Ment Health. 2026 Jan 29;3(1):e0000533. doi: 10.1371/journal.pmen.0000533 (PMC12854475; doi:10.1371/journal.pmen.0000533)
Supplement: S1 Text — (PDF) [file pmen.0000533.s002.pdf]

S1 Text. List of studies excluded at full-text screening, with reasons.

Study	Reason for exclusion
Abedi MR, Vostanis P. Evaluation of quality of life therapy for parents of children with obsessive–compulsive disorders in Iran. Eur Child Adolesc Psychiatry. 2010 Jul;19(7):605–13.	Wrong diagnosis
Ahmad Othman A, Wan Jaafar WM, Zainudin ZN, Yusop YM. Effectiveness of cognitive behavior therapy and acceptance and commitment therapy on depression and anxiety among emerging adults in Malaysia. Curr Psychol. 2024 Mar 1;43(11):9755–72.	Wrong age group
Alavi A, Sharifi B, Ghanizadeh A, Dehbozorgi G. Effectiveness of Cognitive-Behavioral Therapy in Decreasing Suicidal Ideation. Iran J Pediatr. 2013;23(4).	Wrong outcomes
Alavi N, Hirji A, Sutton C, Naeem F. Online CBT Is Effective in Overcoming Cultural and Language Barriers in Patients With Depression. 2016;22(1):2-8.	Wrong age group
Albornoz Y. The effects of group improvisational music therapy on depression in adolescents and adults with substance abuse: a randomized controlled trial**. 2011;20(3):208-224.	Wrong age group
Alipour M, Ghahremani L, Amooee S, Keshavarzi S. The effectiveness of relaxation techniques on depression, anxiety and stress in pregnant women: based on self-efficacy theory. Sci J Kurd Univ Med Sci. 2017;22(3):Pe20-En3.	Wrong age group
Alvi M, Shiri T, Iqbal N, Husain M, Chaudhry I, Shakoor S, et al. Cost-Effectiveness of a Culturally Adapted Manual-Assisted Brief Psychological Intervention for Self-Harm in Pakistan: a Secondary Analysis of the Culturally Adapted Manual-Assisted Brief Psychological Randomized Controlled Trial. 2022;27:65-71.	Wrong age group
Amin R, Iqbal A, Naeem F, Irfan M. Effectiveness of a culturally adapted cognitive behavioural therapy-based guided self-help (CACBT-GSH) intervention to reduce social anxiety and enhance self-esteem in adolescents: a randomized controlled trial from Pakistan. Behav Cogn Psychother. 2020 Sep;48(5):503–14.	Wrong outcomes
Amirpour L., Gharaee B., Birashk B. Efficacy of group transdiagnostic cognitive-behavioral therapy on subclinical paranoia. Iran J Psychiatry Behav Sci. 2018;12(4):e65161.	Wrong age group
Amirpour L., Mirzakhani M., Gharaee B., Birashk B. Efficacy of anxiety-based cognitive behavioral therapy for paranoid ideation in a non-clinical population: A randomized controlled trial. Iran J Psychiatry Behav Sci. 2019;13(2):e81855.	Wrong age group
Araya R., Montgomery A.A., Fritsch R., Gunnell D., Stallard P., Noble S., et al. School-based intervention to improve the mental health of low-income, secondary school students in Santiago, Chile (YPSA): Study protocol for a randomized controlled trial. Trials. 2011;12((Araya) University of Bristol, School of Social and Community Medicine, Oakfield Grove, Bristol BS8 2BN, United Kingdom(Montgomery, Gunnell, Noble) University of Bristol, School of Social and Community Medicine, Canynge Hall, 39 Whatley Road, Bristol BS8):49.	Wrong outcomes
Are A, Olisah V, Bella-Awusah T, Ani C. Controlled clinical trial of teacher-delivered cognitive behavioural therapy (cbt) for adolescents with clinically diagnosed depressive disorder in nigeria. Int J Ment Health. 2021;(Abdulmalik, J., Ani, C., Ajuwon, A. J., Omigbodun, O. (2016) . Effects of problem-solving interventions on aggressive behaviours among primary school pupils in Ibadan, Nigeria . Child and Adolescent Psychiatry and Mental Health, 10 (1), 31 . doi: https:):No-Specified.	Not RCT
Arredondo V, Guerra C. Comparison of the effectiveness of two group interventions for adolescents exposed to interpersonal trauma. 2021; Available from: https://www.cochranelibrary.com/central/doi/10.1002/central/CN-02283141/full	Not LMIC
Askari S., Behroozi N., Abbaspoor Z. The effect of mindfulness-based cognitive-behavioral therapy on premenstrual syndrome. Iran Red Crescent Med J. 2018;20(2):e57538.	Wrong age group

Atigh A, Alizadeh-Zarei M. The effect of cognitive rehabilitation therapy (CRT) on the executive functions of children with autism spectrum disorder (ASD). Chronic Dis J. 2019;7(3):137–47.	Not RCT
Auyeung L, Mo PKH. The efficacy and mechanism of online positive psychological intervention (PPI) on improving well-being among Chinese university students: A pilot study of the best possible self (BPS) intervention. J Happiness Stud Interdiscip Forum Subj Well-Being. 2019;20(8):2525–50.	Wrong age group
Azevedo A., Matos A.P. New treatment for depression with adolescents: Preliminary results. Aten Primaria. 2014;46(Supplement 5):8.	Not LMIC
Baezzat F., Izadifard R., Sadinam M. Effect of cognitive behavioral therapy with problem solving skills training on reduction of symptoms of test anxiety in high school girls. Eur Child Adolesc Psychiatry. 2011;20(SUPPL. 1):S136.	No mental health condition
Baird S. Using Group Interpersonal Psychotherapy to Improve the Well-Being of Adolescent Girls. 2019; Available from: https://www.cochranelibrary.com/central/doi/10.1002/central/CN-01983157/full	Protocol only
Bakhshani N, Lashkaripour K, Sadjadi S. Effectiveness of short term cognitive behavior therapy in patients with generalized anxiety disorder. 2007;7(7):1076-1081.	Wrong age group
Banoğlu K, Korkmazlar Ü. Efficacy of the eye movement desensitization and reprocessing group protocol with children in reducing posttraumatic stress disorder in refugee children. Eur J Trauma Dissociation. 2022 Feb;6(1):100241.	No psychosocial intervention
Benjet C., Kessler R.C., Kazdin A.E., Cuijpers P., Albor Y., Carrasco Tapias N., et al. Study protocol for pragmatic trials of Internet-delivered guided and unguided cognitive behavior therapy for treating depression and anxiety in university students of two Latin American countries: the Yo Puedo Sentirme Bien study. Trials. 2022;23(1):450.	Protocol only
Benjet C. Internet-delivered Cognitive Behavioral Treatment of Depression and Anxiety in Latin American College Students. 2021; Available from: https://www.cochranelibrary.com/central/doi/10.1002/central/CN-02249504/full	Wrong age group
Berger R, Gelkopf M. School-based intervention for the treatment of tsunami-related distress in children: a quasi-randomized controlled trial. 2009;78(6):364-371.	Not RCT
Bernal G., Rivera-Medina C.L., Cumba-Aviles E., Reyes-Rodriguez M.L., Saez-Santiago E., Duarte-Velez Y., et al. Can Cognitive-Behavioral Therapy Be Optimized With Parent Psychoeducation? A Randomized Effectiveness Trial of Adolescents With Major Depression in Puerto Rico. Fam Process. 2019;58(4):832–54.	Not LMIC
Bhana A, Mellins C, Petersen I, Alicea S, Myeza N, Holst H, et al. The VUKA family program: piloting a family-based psychosocial intervention to promote health and mental health among HIV infected early adolescents in South Africa. 2014;26(1):1-11.	No mental health condition
Bilyk BF. The effect of a Pilot Psychological Treatment Program that focuses on thoughts, emotions and behaviors in a sample of Brazilian adolescents with Anorexia Nervosa. 2017; Available from: https://www.cochranelibrary.com/central/doi/10.1002/central/CN-01892105/full	Not RCT
Bingol Caglayan R.H., Demirpence Secinti D. Cognitive behavior therapy for overweight and obese adolescents with psychiatric symptoms: A pilot study. Anadolu Psikiyatri Derg. 2020;21(4):417–22.	Not RCT
Blakeley-Smith AD. Facing Your Fears: adolescents With ASD and Intellectual Disability. 2021; Available from: https://www.cochranelibrary.com/central/doi/10.1002/central/CN-02353128/full	Not LMIC
Bolton P, Lee C, Haroz E, Murray L, Dorsey S, Robinson C, et al. A transdiagnostic community-based mental health treatment for comorbid disorders: development and outcomes of a randomized controlled trial among Burmese refugees in Thailand. 2014;11(11):e1001757.	Wrong age group
Bong S.H., Won G.H., Choi T.Y. Effects of cognitive-behavioral therapy based music therapy in korean adolescents with smartphone and internet addiction. Psychiatry Investig. 2021;18(2):110–7.	Not LMIC

Brathwaite R, Ssewamala FM, Sensoy Bahar O, McKay MM, Neilands TB, Namatovu P, et al. The longitudinal impact of an evidence-based multiple family group intervention (Amaka Amasanyufu) on oppositional defiant disorder and impaired functioning among children in Uganda: analysis of a cluster randomized trial from the SMART Africa-Uganda scale-up study (2016–2022). J Child Psychol Psychiatry. 2022;63(11):1252–60.	Wrong diagnosis
Brown F.L., Steen F., Taha K., Aoun M., Bryant R.A., Jordans M.J.D., et al. Early Adolescent Skills for Emotions (EASE) intervention for the treatment of psychological distress in adolescents: Study protocol for randomised controlled trials in Lebanon and Jordan. Trials. 2019;20(1):545.	Protocol only
Brückner B, Huss M. A randomized, waiting list controlled, multi-center study to evaluate START (Stress-Trauma Symptoms-Arousal-Regulation-Treatment) training for young refugees with traumatic stress related disorders. 2020; Available from: https://www.cochranelibrary.com/central/doi/10.1002/central/CN-02168821/full	Not LMIC
Bryant R.A., Bawaneh A., Awwad M., Al-Hayek H., Giardinelli L., Whitney C., et al. Effectiveness of a brief group behavioral intervention for common mental disorders in Syrian refugees in Jordan: A randomized controlled trial. PLoS Med. 2022;19(3):e1003949.	Wrong age group
Budde H, Akko DP. The impact of an exercise training intervention on cortisol levels and Post-Traumatic Stress Disorder in Congolese refugees: study protocol for a randomized control trial. 2018; Available from: https://www.cochranelibrary.com/central/doi/10.1002/central/CN-01899135/full	No psychosocial intervention
Bustamante Loyola J, Perez Retamal M, Mendiburo-Seguel A, Guedeney A, Salinas Gonzalez R, Munoz L, et al. The Impact of an Interactive Guidance Intervention on Sustained Social Withdrawal in Preterm Infants in Chile: randomized Controlled Trial. 2022;10. Available from: https://www.cochranelibrary.com/central/doi/10.1002/central/CN-02394923/full	Not LMIC
Byansi W., Ssewamala F.M., Neilands T.B., Sensoy Bahar O., Nabunya P., Namuwonge F., et al. The Short-Term Impact of a Combination Intervention on Depressive Symptoms Among School-Going Adolescent Girls in Southwestern Uganda: The Suubi4Her Cluster Randomized Trial. J Adolesc Health. 2022;71(3):301–7.	No mental health condition
Byansi W. Utilization patterns of community-based mental health services among school-going adolescent girls in southwestern Uganda. Diss Abstr Int Sect B Sci Eng. 2022;83(3-B):No-Specified.	Not RCT
Casella C.B., Zuccolo P.F., Sugaya L., de Souza A.S., Otoch L., Alarcao F., et al. Brief internet-delivered cognitive-behavioural intervention for children and adolescents with symptoms of anxiety and depression during the COVID-19 pandemic: a randomised controlled trial protocol. Trials. 2022;23(1):899.	Protocol only
Catani C, Kohiladevy M, Ruf M, Schauer E, Elbert T, Neuner F. Treating children traumatized by war and Tsunami: A comparison between exposure therapy and meditation-relaxation in North-East Sri Lanka. BMC Psychiatry. 2009 Dec;9(1):22.	Not a group intervention
Cavazos-Rehg P, Byansi W, Doroshenko C, Neilands TB, Anako N, Sensoy Bahar O, et al. Evaluating potential mediators for the impact of a family-based economic intervention (Suubi+Adherence) on the mental health of adolescents living with HIV in Uganda. Soc Sci Med. 2021;280(Ahmed, C.V., Jolly, P., Padilla, L., Malinga, M., Harris, C., Mthethwa, N., Preko, P. (2017). A qualitative analysis of the barriers to antiretroviral therapy initiation among children 2 to 18 months of age in Swaziland. Afr. J. AIDS Res., 16, 4, 321-32).	No psychosocial intervention
Chan N.Y., Li S.X., Zhang J., Lam S.P., Yu M.M.W., Kwok P.L., et al. Can we prevent insomnia?A brief cognitive behavioral therapy in at-risk adolescents. Sleep Med. 2019;64(Supplement 1):S61.	Not LMIC
Chan S.K.C., Zhang D., Bogels S.M., Chan C.S., Lai K.Y.C., Lo H.H.M., et al. Effects of a mindfulness-based intervention (MYmind) for children with ADHD and their parents: Protocol for a randomised controlled trial. BMJ Open. 2018;8(11):e022514.	Not LMIC
Chang W, Chan G, Jim O, Lau E, Hui C, Chan S, et al. Optimal duration of an early intervention programme for first-episode psychosis: randomised controlled trial. 2015;206(6):492-500.	Not LMIC
Chang W, Kwong V, Chan G, Jim O, Lau E, Hui C, et al. Prediction of motivational impairment: 12-month follow-up of the randomized-controlled trial on extended early intervention for first-episode psychosis. 2017;41:37-41.	Wrong age group

CHEN J. The Effect of G-CBT on the Patients With AN : a Randomized Controlled Trial. 2018; Available from: https://www.cochranelibrary.com/central/doi/10.1002/central/CN-01663598/full	Wrong age group
CHEN J. The Effect of G-DBT on the Patients With BN : a Multicenter Randomized Controlled Study. 2018; Available from: https://www.cochranelibrary.com/central/doi/10.1002/central/CN-01483698/full	Wrong age group
Chen QiuZhen. Psychological intervention of anxiety in postgraduate students during academic activities. China Trop Med. 2007;7(8):1495–1299.	Wrong age group
Chen X., Zhang X., Zhu X., Wang G. Efficacy of an Internet-Based Intervention for Subclinical Depression (MoodBox) in China: Study Protocol for a Randomized Controlled Trial. Front Psychiatry. 2020;11((Chen, Zhang, Zhu, Wang) Beijing Key Laboratory of Mental Disorders, The National Clinical Research Center for Mental Disorders, Beijing Anding Hospital, Capital Medical University, Beijing, China(Chen, Zhang, Zhu, Wang) Advanced Innovation Center for Hum):585920.	Protocol only
Chen X, Hannibal N, Gold C. Randomized Trial of Group Music Therapy With Chinese Prisoners: impact on Anxiety, Depression, and Self-Esteem. 2016;60(9):1064-1081.	Wrong age group
Chinoda S, Mutsinze A, Simms V, Beji-Chauke R, Verhey R, Robinson J, et al. Effectiveness of a peer-led adolescent mental health intervention on HIV virological suppression and mental health in Zimbabwe: protocol of a cluster-randomised trial. Glob Ment Health. 2020;7:e23.	No mental health condition
Chiumento A, Hamdani S, Khan M, Dawson K, Bryant R, Sijbrandij M, et al. Evaluating effectiveness and cost-effectiveness of a group psychological intervention using cognitive behavioural strategies for women with common mental disorders in conflict-affected rural Pakistan: study protocol for a randomised controlled trial. 2017;18(1):190.	Wrong age group
Chowdhary N, Anand A, Dimidjian S, Shinde S, Weobong B, Balaji M, et al. The Healthy Activity Program lay counsellor delivered treatment for severe depression in India: systematic development and randomised evaluation. 2016;208(4):381-388.	Not RCT
Coelho LF, Barbosa DLF, Rizzutti S, Bueno OFA, Miranda MC. Group cognitive behavioral therapy for children and adolescents with ADHD. Psicol Reflexao E Crit. 2017;30(AACAP. (2007). Practice parameter for the assessment and treatment of children and adolescents with attention-deficit/hyperactivity disorder, (July), 894-921. http://doi.org/10.1097/chi.0b013e318054e724.Abadie, A., Drukker, D., Herr, J. L., Imbens, G. W).	Not RCT
Costescu CA, Vanderborght B, Robotics and Multibody Mechanics Research Group, Vrije Universiteit Brussel, bram.vanderborght@vub.ac.be, David DO, Department of Clinical Psychology and Psychotherapy, Babes-Bolyai University, Cluj-Napoca, Romania, danieldavid@psychology.ro. Robot-Enhanced CBT for dysfunctional emotions in social situations for children with ASD. J Evid-Based Psychother. 2017 Sep 1;17(2):119–32.	Wrong diagnosis
Cui L, He F, Han Z, Yang R, Xiao J, Oei TPS. A brief group cognitive-behavioral program for the prevention of depressive symptoms in Chinese college students. Int J Group Psychother. 2016;66(2):291–307.	Wrong age group
Damra JKM, Nassar YH, Ghabri TMF. Trauma-focused cognitive behavioral therapy: Cultural adaptations for application in Jordanian culture. Couns Psychol Q. 2014 Jul 3;27(3):308–23.	Wrong outcomes
Dawson K, Joscelyne A, Meijer C, Steel Z, Silove D, Bryant RA. A controlled trial of trauma-focused therapy versus problem-solving in Islamic children affected by civil conflict and disaster in Aceh, Indonesia. Aust N Z J Psychiatry. 2018 Mar;52(3):253–61.	Not a group intervention
Deakin E.K., Nunes M.L.T. Effectiveness of child psychoanalytic psychotherapy in a clinical outpatient setting. J Child Psychother. 2009;35(3):290–301.	Not RCT
Demenech LM. GRAU: group-based therapy for anxiety reduction among university students. 2020; Available from: https://www.cochranelibrary.com/central/doi/10.1002/central/CN-02173824/full	Wrong age group
DEMIRKOL H. Online Psychosocial Intervention for Nursing Students Who Experienced Intimate Partner Abuse in Turkey. 2022; Available from: https://www.cochranelibrary.com/central/doi/10.1002/central/CN-02398905/full	Wrong age group

Dhital R., Shibanuma A., Miyaguchi M., Kiriya J., Jimba M. Effect of psycho-social support by teachers on improving mental health and hope of adolescents in an earthquake-affected district in Nepal: A cluster randomized controlled trial. PLoS ONE. 2019;14(10):e0223046.	No mental health condition
Do R., Lee S., Kim J.-S., Cho M., Shin H., Jang M., et al. Effectiveness and dissemination of computer-based cognitive behavioral therapy for depressed adolescents: Effective and accessible to whom? J Affect Disord. 2021;282((Do) Department of Clinical Medical Sciences, Seoul National University, College of Medicine, Seoul, South Korea(Lee) Biomedical Research Institute, Seoul National University Hospital, Seoul, South Korea(Kim, Cho, Shin, Jang) Department of Child and Adole):885–93.	Not LMIC
Dogra A, Veeraraghavan V. A study of psychological intervention of children with aggressive conduct disorder. Indian J Clin Psychol. 1994;21(1):28–32.	Not RCT
Donenberg G.R., Fitts J., Ingabire C., Nsanzimana S., Fabri M., Emerson E., et al. Results of the Kigali Imbereheza Project: A 2-Arm Individually Randomized Trial of TI-CBT Enhanced to Address ART Adherence and Mental Health for Rwandan Youth Living With HIV. J Acquir Immune Defic Syndr. 2022;90(1):69–78.	No mental health condition
Dorsey S, Gray C, Wasonga A, Amanya C, Weiner B, Belden C, et al. Advancing successful implementation of task-shifted mental health care in low-resource settings (BASIC): protocol for a stepped wedge cluster randomized trial. 2020;20(1):10.	Protocol only
Duan C.-C., Yu J.-L., Tao J., Zhang C., Zhang D., Zeng X., et al. Internet-based cognitive therapy for women with antenatal depressive symptoms during the COVID-19 pandemic: protocol for a multi-center randomized controlled trial across China. Trials. 2022;23(1):797.	Wrong age group
Ebesutani CK, Helmi K, Fierstein M, Taghizadeh ME, Chorpita BF. A Pilot Study of Modular Cognitive-Behavioral Therapy and Cognitive-Behavioral Hypnotherapy for Treating Anxiety in Iranian Girls. Int J Cogn Ther. 2016 Mar;9(1):13–37.	Not a group intervention
Edeh N.I., Ugwoke E.O., Anaele E.N., Madusaba B.M., Naboth-Odums A., Isiwu E.A., et al. Supporting business educators and students against COVID-19 trauma using trauma-focused cognitive behavioral therapy. Med U S. 2022;101(14):e29133.	Wrong age group
Egbe CI, Ugwuanyi LT, Ede MO, Agbigwe IB, Onuorah AR, Okon OE, et al. Cognitive Behavioural Play Therapy for Social Anxiety Disorders (SADs) in Children with Speech Impairments. J Ration-Emotive Cogn-Behav Ther. 2023 Mar;41(1):24–44.	Wrong outcomes
Egbegi DR, Bella-Awusah T, Omigbodun O, Ani C. A controlled trial of Cognitive Behavioural Therapy-based strategies for insomnia among in-school adolescents in southern Nigeria. Child Adolesc Psychiatry Ment Health. 2021 Dec;15(1):52.	Wrong diagnosis
Egenti NT, Ede MO, Nwokenna EN, Oforka T, Nwokeoma BN, Mezieobi DI, et al. Randomized controlled evaluation of the effect of music therapy with cognitive-behavioral therapy on social anxiety symptoms: Retracted. Medicine (Baltimore). 2019 Aug;98(32):e16495.	Wrong outcomes
El-Tellawy MM, Ahmad AR, Saad K, Alruwaili TAM, AbdelMoneim IM, Shaaban I, et al. Effect of hyperbaric oxygen therapy and Tomatis sound therapy in children with autism spectrum disorder. Prog Neuropsychopharmacol Biol Psychiatry. 2022 Mar;113:110457.	Wrong diagnosis
Ertl V, Pfeiffer A, Schauer E, Elbert T, Neuner F. Community-Implemented Trauma Therapy for Former Child Soldiers in Northern Uganda: A Randomized Controlled Trial. JAMA. 2011 Aug 3;306(5):503.	Not a group intervention
Ezegbe B.N., Eseadi C., Onyemaechi M., Igbo J.N., Anyanwu J.I., Ede K.R., et al. Impacts of cognitive-behavioral intervention on anxiety and depression among social science education students. Med U S. 2019;98(15):e14935.	Wrong age group
Fereydouni S., Forstmeier S. An Islamic Form of Logotherapy in the Treatment of Depression, Anxiety and Stress Symptoms in University Students in Iran. J Relig Health. 2022;61(1):139–57.	Wrong age group
Givi H.G., Imani H., Agh A., Rik N.M., Mehrabadi S. Efficiency of computerized cognitive behavioral therapy versus clinical intervention for the treatment of major depression. Koomesh. 2012;13(2):218–24.	Not a group intervention

Gomes B, Abreu L, Brietzke E, Caetano S, Kleinman A, Nery F, et al. A randomized controlled trial of cognitive behavioral group therapy for bipolar disorder. 2011;80(3):144-150.	Wrong age group
Gormez V., Kilic H.N., Orengul A.C., Demir M.N., Mert E.B., Makhlouta B., et al. Evaluation of a school-based, teacher-delivered psychological intervention group program for trauma-affected Syrian refugee children in Istanbul, Turkey. Psychiatry Clin Psychopharmacol. 2017;27(2):125–31.	Not RCT
Grigaliuniene V., Vaskelyte A., Bulikaite V. Effectiveness of dialectical behaviour therapy for children suffering from emotional and behavioural disorders. Arch Dis Child. 2014;99(SUPPL. 2):A559.	Not RCT
Gureje O, Oladeji BD, Kola L, Bello T, Ayinde O, Faregh N, et al. Effect of intervention delivered by frontline maternal care providers to improve outcome and parenting skills among adolescents with perinatal depression in Nigeria (the RAPiD study): A cluster randomized controlled trial. J Affect Disord. 2022 Sep;312:169–76.	Not a group intervention
Haack LM, Araujo EA, Meza J, Friedman LM, Spiess M, Alcaraz Beltrán DK, et al. Can School Mental Health Providers Deliver Psychosocial Treatment Improving Youth Attention and Behavior in Mexico? A Pilot Randomized Controlled Trial of CLS-FUERTE. J Atten Disord. 2021 Dec;25(14):2083–97.	Not a treatment intervention
Hamdani S.U., Huma Z.-E., Malik A., Nizami A.T., Baneen U.U., Suleman N., et al. Improving psychosocial distress for young adolescents in rural schools of Pakistan: study protocol of a cluster randomised controlled trial. BMJ Open. 2022;12(9):e063607.	Protocol only
Hamdieh M, Taraghijah S. The effect of Cognitive Spiritual Group Therapy (CSGT) in depression. Pejouhandeh. 2008;13(5):Pe383-En2.	Wrong age group
Hamedi V., Hamid N., Beshlideh K., Marashi S.A., Sheikh Shabani S.E.H. Effectiveness of Conventional Cognitive-Behavioral Therapy and Its Computerized Version on Reduction in Pain Intensity, Depression, Anger, and Anxiety in Children with Cancer: A Randomized, Controlled Trial. Iran J Psychiatry Behav Sci. 2020;14(4):83110.	No mental health condition
Hamid N, Molajegh R, Bashlideh K, Shehniyailagh M. The comparison of effectiveness of dialectical behavioral therapy (DBT) and schema therapy (ST) in reducing the severity of clinical symptoms (disruptive communication, emotional deregulation and behavioral deregulation) of borderline personality disorde. 2020;14(2):1354-1363.	Wrong age group
Hanani A. Effect of cognitive behavioral therapy program on mental health status among medical student in Palestine during COVID-19 pandemic. 2021; Available from: https://www.cochranelibrary.com/central/doi/10.1002/central/CN-02352402/full	Wrong age group
Hasanovic M., Husanovic J., Srabovic S., Haskic E., Lukic D., Jaganjac A. Psychosocial assistance project decreased severity of posttraumatic stress disorder symptoms and and depressivenes amongst school adolescents in post-war bosnia herzegovina. Eur Psychiatry. 2012;27(SUPPL. 1).	Not RCT
Hasanovic M, Srabovic S, Rasidovic M, Sehovic M, Hasanbasic E, Husanovic J, et al. Psychosocial assistance project decreases posttraumatic stress disorder and depression amongst primary and secondary schools students in post-war Bosnia-Herzegovina. Acta Medica Acad. 2011;40(2):122–31.	Not RCT
Hazelton M. Effects of mindfulness-based stress reduction (MBSR) on stress, depression, self-esteem and mindfulness in Thai nursing students. 2014; Available from: https://www.cochranelibrary.com/central/doi/10.1002/central/CN-01845751/full	No mental health condition
He HL, Zhang M, Gu CZ, Xue RR, Liu HX, Gao CF, et al. Effect of cognitive behavioral therapy on improving the cognitive function in major and minor depression. J Nerv Ment Dis. 2019;207(4):232–8.	Wrong age group
He Y, Yang L, Zhu X, Wu B, Zhang S, Qian C, et al. Mental Health Chatbot for Young Adults With Depressive Symptoms During the COVID-19 Pandemic: Single-Blind, Three-Arm Randomized Controlled Trial. J Med Internet Res. 2022 Nov 21;24(11):e40719.	Not a group intervention
Hemyari C., Zomorodian K., Shojaee M., Sahraian A., Dolatshahi B. The effect of personality traits on cognitive behavioral therapy outcomes in student pharmacists with rat phobia: A randomized clinical trial. Iran J Med Sci. 2021;46(1):23–31.	Wrong age group

Hinsberger M., Holtzhausen L., Sommer J., Kaminer D., Elbert T., Seedat S., et al. Long-term effects of psychotherapy in a context of continuous community and gang violence: changes in aggressive attitude in high-risk South African adolescents. Behav Cogn Psychother. 2020;48(1):1–13.	Wrong age group
Högberg G, Hällström T. Mood Regulation Focused CBT Based on Memory Reconsolidation, Reduced Suicidal Ideation and Depression in Youth in a Randomised Controlled Study. 2018;15(5). Available from: https://www.cochranelibrary.com/central/doi/10.1002/central/CN-01649635/full	Not LMIC
Hong J.S., Kim S.M., Kang K.D., Han D.H., Kim J.S., Hwang H., et al. Effect of physical exercise intervention on mood and frontal alpha asymmetry in internet gaming disorder: Physical exercise intervention for IGD. Ment Health Phys Act. 2020;18((Hong, Kim, Kang, Han, Hwang, Min, Lee) Department of Psychiatry, Chung-Ang University College of Medicine, 84 Heukseok-ro, Dongjak-gu, Seoul 06974, South Korea(Kim) Anseong Elementary School, 13 Hyesan-ro, Anseong-si, Gyeonggi-do 17587, South Korea(Choi)):100318.	Not LMIC
Hong JS, Kim SM, Kang KD, Han DH, Kim JS, Hwang H, et al. Effect of physical exercise intervention on mood and frontal alpha asymmetry in internet gaming disorder. Ment Health Phys Act. 2020;18(American Psychiatric Association. (2013). Diagnostic and statistical manual of mental disorders (DSM-5). Washington, DC: American Psychiatric Association.2013-14907-000Barnhofer, T., Duggan, D., Crane, C., Hepburn, S., Fennell, M.J., Williams, J.M. (200).	Not LMIC
Huang Y.-H., Chung C.-Y., Ou H.-Y., Tzang R.-F., Huang K.-Y., Liu H.-C., et al. Treatment effects of combining social skill training and parent training in Taiwanese children with attention deficit hyperactivity disorder. J Formos Med Assoc. 2015;114(3):260–7.	Not LMIC
Humphreys K, McGoron L, Sheridan M, McLaughlin K, Fox N, Nelson C, et al. High-Quality Foster Care Mitigates Callous-Unemotional Traits Following Early Deprivation in Boys: a Randomized Controlled Trial. 2015;54(12):977-983.	No mental health condition
Husain N, Afsar S, Ara J, Fayyaz H, Rahman R, Tomenson B, et al. Brief psychological intervention after self-harm: randomised controlled trial from Pakistan. 2014;204(6):462-470.	Wrong age group
Husain N, Chaudhry N, Fatima B, Husain M, Amin R, Chaudhry I, et al. Antidepressant and group psychosocial treatment for depression: a rater blind exploratory RCT from a low income country. 2014;42(6):693-705.	Wrong age group
Husain N., Kiran T., Fatima B., Chaudhry I.B., Saeed Q., Masood S.N., et al. Development and assessment of a mobile phone-based intervention to reduce maternal depression and improve child health. Eur Psychiatry. 2016;33(SUPPL.):S608–9.	Not RCT
Husain N., Zulqernain F., Carter L.-A., Chaudhry I.B., Fatima B., Kiran T., et al. Treatment of maternal depression in urban slums of Karachi, Pakistan: A randomized controlled trial (RCT) of an integrated maternal psychological and early child development intervention. Asian J Psychiatry. 2017;29((Husain, Chaudhry) Division of Psychology and Mental Health, School of Health Sciences, University of Manchester, United Kingdom(Husain, Zulqernain, Chaudhry, Fatima, Kiran, Chaudhry, Naeem, Jafri, Lunat, Haq, Husain) Pakistan Institute of Living&Learni):63–70.	Wrong age group
Husain N, Kiran T, Fatima B, Chaudhry IB, Husain M, Shah S, et al. An integrated parenting intervention for maternal depression and child development in a low-resource setting: Cluster randomized controlled trial. Depress Anxiety. 2021;38(9):925–39.	Wrong age group
Hyun M.-S., Nam K.A., Kim M.-A. Randomized Controlled Trial of a Cognitive-Behavioral Therapy for At-risk Korean Male Adolescents. Arch Psychiatr Nurs. 2010;24(3):202–11.	Not LMIC
Iftene F, Predescu E, Stefan S, David D. Rational-emotive and cognitive-behavior therapy (REBT/CBT) versus pharmacotherapy versus REBT/CBT plus pharmacotherapy in the treatment of major depressive disorder in youth; A randomized clinical trial. Psychiatry Res. 2015 Feb;225(3):687–94.	Clinical setting
Irfan M. Culturally Adapted CBT Based Guided Self-Help in Patients With Postnatal Depression. 2020; Available from: https://www.cochranelibrary.com/central/doi/10.1002/central/CN-02089786/full	Wrong age group
Izadi-Mazidi M., Davoudi I., Mehrabizadeh M. Effect of group cognitive-behavioral therapy on health-related quality of life in females with premenstrual syndrome. Iran J Psychiatry Behav Sci. 2016;10(1):e4961.	Wrong age group

Jaberghaderi N, Greenwald R, Rubin A, Zand S, Dolatabadi S. A comparison of CBT and EMDR for sexually-abused Iranian girls. 2004;11(5):358-368.	Not RCT
Jacob N, Neuner F, Maedl A, Schaal S, Elbert T. Dissemination of psychotherapy for trauma spectrum disorders in postconflict settings: a randomized controlled trial in Rwanda. 2014;83(6):354-363.	Wrong age group
Jordans M.J., Tol W.A., Ndayisaba A., Komproe I.H. A controlled evaluation of a brief parenting psychoeducation intervention in Burundi. Soc Psychiatry Psychiatr Epidemiol. 2013;48(11):1851–9.	Not RCT
Jordans M. Early Adolescent Skills for Emotions (EASE) for young adolescents in Lebanon. 2019; Available from: https://www.cochranelibrary.com/central/doi/10.1002/central/CN-01968813/full	Protocol only
Jordans M. Feasibility trial of a psychological intervention with young adolescents in Lebanon. 2022; Available from: https://www.cochranelibrary.com/central/doi/10.1002/central/CN-02469801/full	Protocol only
Kaminer D. A randomised controlled trial of TF-CBT in South African adolecents. 2020; Available from: https://www.cochranelibrary.com/central/doi/10.1002/central/CN-02238922/full	Not a group intervention
Kananian S, Soltani Y, Hinton D, Stangier U. Culturally Adapted Cognitive Behavioral Therapy Plus Problem Management (CA-CBT+) With Afghan Refugees: a Randomized Controlled Pilot Study. 2020;33(6):928-938.	Not LMIC
Kanuri N, Newman MG, Ruzek JI, Kuhn E, Manjula M, Jones M, et al. The Feasibility, Acceptability, and Efficacy of Delivering Internet-Based Self-Help and Guided Self-Help Interventions for Generalized Anxiety Disorder to Indian University Students: Design of a Randomized Controlled Trial. JMIR Res Protoc. 2015;4(4):e136.	Protocol only
Kazempour V, Ebrahimi H, Asghari Jafarabadi M, Nourazar SG, Zamani H. The Effect of Group Cognitive Behavioral Therapy on Cognitive Emotion Regulation Strategies of Adolescents with Bipolar Disorder During Their Euthymic Phase: A Randomized, Controlled Trial. Iran Red Crescent Med J [Internet]. 2018 May 5 [cited 2024 May 13];In Press(In Press). Available from: https://archive.ircmj.com/article/20/s1/ircmj-20-S1-61555.pdf	Wrong diagnosis
Klos MC, Escoredo M, Joerin A, Lemos VN, Rauws M, Bunge EL. Artificial Intelligence-Based Chatbot for Anxiety and Depression in University Students: Pilot Randomized Controlled Trial. JMIR Form Res. 2021;5(8):e20678.	Wrong age group
Knaevelsrud C, Brand J, Lange A, Ruwaard J, Wagner B. Web-based psychotherapy for posttraumatic stress disorder in war-traumatized Arab patients: randomized controlled trial. 2015;17(3):e71.	Wrong age group
Knijnik D, Kapczinski F, Chachamovich E, Margis R, Eizirik C. Psychodynamic group treatment for generalized social phobia. 2004;26(2):77-81.	Wrong age group
Koebach A., Carleial S., Elbert T., Schmitt S., Robjant K. Treating Trauma and Aggression With Narrative Exposure Therapy in Former Child and Adult Soldiers: A Randomized Controlled Trial in Eastern DR Congo. J Consult Clin Psychol. 2021;89(3):143–55.	Wrong age group
Kumar M., Huang K.-Y., Othieno C., Wamalwa D., Hoagwood K., Unutzer J., et al. Implementing combined WHO mhGAP and adapted group interpersonal psychotherapy to address depression and mental health needs of pregnant adolescents in Kenyan primary health care settings (INSPIRE): a study protocol for pilot feasibility trial of the integ. Pilot Feasibility Stud. 2020;6(1):136.	Protocol only
Kumuyi D.O., Akinnawo E.O., Akpunne B.C., Akintola A.A., Onisile D.F., Aniemeka O.O. Effectiveness of cognitive behavioural therapy and social skills training in management of conduct disorder. South Afr J Psychiatry. 2022;28((Kumuyi, Akinnawo, Akpunne, Akintola, Aniemeka) Department of Behavioural Studies, Faculty of Social Sciences, Redeemer's University, Osun State, Ede, Nigeria(Onisile) Department of Nursing Science, Faculty of Basic Medical Sciences, Redeemer's University):a1737.	Not RCT
Lan YT, Liu XP, Fang HS. Randomized control study of the effects of executive function training on peer difficulties of children with attention-deficit/hyperactivity disorder C subtype. Appl Neuropsychol Child. 2020 Jan 2;9(1):41–55.	Wrong diagnosis

Lau W.-Y., Chan C.K.-Y., Li J.C.-H., Au T.K.-F. Effectiveness of group cognitive-behavioral treatment for childhood anxiety in community clinics. Behav Res Ther. 2010;48(11):1067–77.	Not LMIC
Le S, Sijing C. Effectiveness of eCBT-I on Enhancing Depression and Insomnia Outcome in Chinese Youths With Both Diagnoses. 2021; Available from: https://www.cochranelibrary.com/central/doi/10.1002/central/CN-02438967/full	Protocol only
Li G, Dai XY. Control study of cognitive-behavior therapy in adolescents with Internet addiction disorder. Chin Ment Health J. 2009;23(7):457–70.	Wrong outcomes
Li H, Wang S. The role of cognitive distortion in online game addiction among Chinese adolescents. Child Youth Serv Rev. 2013 Sep;35(9):1468–75.	Wrong diagnosis
Li J, Liu Y. Intervention Effect of the Video Health Education Model Based on Solution-Focused Theory on Adolescents' Mental Health during the COVID-19 Pandemic. Iran J Public Health [Internet]. 2021 Oct 26 [cited 2024 May 13]; Available from: https://publish.kne-publishing.com/index.php/ijph/article/view/7574	Not a group intervention
Liang L., Feng L., Zheng X., Wu Y., Zhang C., Li J. Effect of dialectical behavior group therapy on the anxiety and depression of medical students under the normalization of epidemic prevention and control for the COVID-19 epidemic: a randomized study. Ann Palliat Med. 2021;10(10):10591–9.	Wrong age group
Lin TJ, Ko HC, Wu JYW, Oei TP, Lane HY, Chen CH. The effectiveness of dialectical behavior therapy skills training group vs. cognitive therapy group on reducing depression and suicide attempts for borderline personality disorder in Taiwan. Arch Suicide Res. 2019;23(1):82–99.	Not LMIC
Lin X, Fang X, Chi P, Li X, Chen W, Heath MA. Grief-processing-based psychological intervention for children orphaned by AIDS in central China: A pilot study. Sch Psychol Int. 2014;35(6):609–26.	Not RCT
Lu R, Zhou Y, Wu Q, Peng X, Dong J, Zhu Z, et al. The effects of mindfulness training on suicide ideation among left-behind children in China: a randomized controlled trial. 2019;45(3):371-379.	No mental health condition
Maalouf F.T., Alrojolah L., Ghandour L., Afifi R., Dirani L.A., Barrett P., et al. Building Emotional Resilience in Youth in Lebanon: a School-Based Randomized Controlled Trial of the FRIENDS Intervention. Prev Sci Off J Soc Prev Res. 2020;21(5):650–60.	No mental health condition
Mahmood Z., Kelsven S., Cadenhead K., Wyckoff J., Reyes-Madrigal F., de la Fuente-Sandoval C., et al. Compensatory Cognitive Training for Latino Youth at Clinical High Risk for Psychosis: Study Protocol for a Randomized Controlled Trial. Front Psychiatry. 2020;10((Mahmood, Kelsven) San Diego State University/University of California, San Diego Joint Doctoral Program in Clinical Psychology, San Diego, CA, United States(Mahmood) Research Service, VA San Diego Healthcare System, San Diego, CA, United States(Cadenhead):951.	Not LMIC
Mahmood Z, Kelsven S, Cadenhead K, Wyckoff J, Reyes-Madrigal F, de la Fuente-Sandoval C, et al. Compensatory Cognitive Training for Latino Youth at Clinical High Risk for Psychosis: Study Protocol for a Randomized Controlled Trial. Front Psychiatry. 2019;10(101545006):951.	Not LMIC
Manjula M, Prasadarao P, Kumaraiah V, Raguram R. Temporal patterns of change in panic disorder during cognitive behaviour therapy: an Indian study. 2014;42(5):513-525.	Wrong age group
Marmar C. Trial of Mental Health Treatment for Darfur Refugees in Cairo. 2009; Available from: https://www.cochranelibrary.com/central/doi/10.1002/central/CN-01500408/full	Wrong age group
Martinez V., Martinez P., Vohringer P.A., Araya R., Rojas G. Computer-assisted cognitive-behavioral therapy for adolescent depression in primary care clinics in Santiago, Chile (YPSA-M): Study protocol for a randomized controlled trial. Trials. 2014;15(1):309.	Not LMIC
Martínez-Nahuel V. Computer-Assisted Cognitive-Behavioral Therapy for Adolescent Depression. 2013; Available from: https://www.cochranelibrary.com/central/doi/10.1002/central/CN-02033805/full	Not LMIC
Masror E.N., Rad M.M., Farrokhi N., Fallah R., Ghahari S. Efficacy of dialectical behaviour techniques (DBT) in reduction of anxiety of students in Tehran- Iran. J Glob Pharma Technol. 2016;8(12):196–201.	Not RCT

Matijasevich A. Parent-mediated Social-communication Therapy for Young Children Living in Poverty in Brazil. 2021; Available from: https://www.cochranelibrary.com/central/doi/10.1002/central/CN-02307082/full	Wrong outcomes
McMullen J., O'Callaghan P., Shannon C., Black A., Eakin J. Group trauma-focused cognitive-behavioural therapy with former child soldiers and other war-affected boys in the DR Congo: a randomised controlled trial. J Child Psychol Psychiatry. 2013;54(11):1231–41.	No mental health condition
Meng F, Han H, Luo J, Liu J, Liu Z, Tang Y, et al. Efficacy of cognitive behavioural therapy with medication for patients with obsessive-compulsive disorder: a multicentre randomised controlled trial in China. 2019;253:184-192.	Wrong age group
Mojahed A, Zaheri Y, Firoozkoohi Moqaddam M. Effectiveness of group psychodrama on aggression and social anxiety of children with attention-deficit/hyperactivity disorder: A randomized clinical trial. Arts Psychother. 2021 Apr;73:101756.	Wrong diagnosis
Murray LK, Skavenski S, Kane JC, Mayeya J, Dorsey S, Cohen JA, et al. Effectiveness of Trauma-Focused Cognitive Behavioral Therapy Among Trauma-Affected Children in Lusaka, Zambia: A Randomized Clinical Trial. JAMA Pediatr. 2015 Aug 1;169(8):761.	Not a group intervention
Nasseh A. Treating depression: Combining group cognitive-behavior therapy (CBT), parent skill training (PST), and joint parent-adolescent sessions (JPAS). J Iran Psychol. 2007;3(12):323–32.	Not RCT
Naveed A, Masood S. A comparison of group art therapy in decreasing the depression level of leukamia paediatric patients. 2020;67(SUPPL 4). Available from: https://www.cochranelibrary.com/central/doi/10.1002/central/CN-02244149/full	Not RCT
Neufeld CB, Palma PC, Caetano KAS, Brust-Renck PG, Curtiss J, Hofmann SG. A randomized clinical trial of group and individual Cognitive-Behavioral Therapy approaches for Social Anxiety Disorder. Int J Clin Health Psychol IJCHP. 2020;20(1):29–37.	Wrong age group
Neuner F, Onyut P, Ertl V, Odenwald M, Schauer E, Elbert T. Treatment of posttraumatic stress disorder by trained lay counselors in an African refugee settlement: a randomized controlled trial. 2008;76(4):686-694.	Wrong age group
Niemi M, Kiel S, Allebeck P, Hoan le T. Community-based intervention for depression management at the primary care level in Ha Nam Province, Vietnam: a cluster-randomised controlled trial. 2016;21(5):654-661.	Wrong age group
Nunez D., Gaete J., Meza D., Andaur J., Robinson J. Testing the Effectiveness of a Blended Intervention to Reduce Suicidal Ideation among School Adolescents in Chile: A Protocol for a Cluster Randomized Controlled Trial. Int J Environ Res Public Health. 2022;19(7):3947.	Not LMIC
Nunez D. Effectiveness of a CBT Online-based Program to Reduce Suicide Ideation Among School Adolescents. 2022; Available from: https://www.cochranelibrary.com/central/doi/10.1002/central/CN-02367501/full	Not LMIC
O'Donnell LA, Weintraub MJ, Ellis AJ, Axelson DA, Kowatch RA, Schneck CD, et al. A Randomized Comparison of Two Psychosocial Interventions on Family Functioning in Adolescents with Bipolar Disorder. Fam Process. 2020;59(2):376–89.	Not LMIC
Obiweluozo PE, Ede MO, Onwurah CN, Uzodinma UE, Dike IC, Ejiofor JN. Impact of cognitive behavioural play therapy on social anxiety among school children with stuttering deficit: A cluster randomised trial with three months follow-up. Medicine (Baltimore). 2021 May 14;100(19):e24350.	Wrong diagnosis
Oh Y, Joung YS, Jang B, Yoo JH, Song J, Kim J, et al. Efficacy of hippotherapy versus pharmacotherapy in attention-deficit/hyperactivity disorder: A randomized clinical trial. J Altern Complement Med. 2018;24(5):463–71.	Not LMIC
Olowokere AE, Okanlawon FA. Improving vulnerable school children's psychosocial health outcomes through resilience-based training and peer-support activities: A comparative prospective study. Vulnerable Child Youth Stud. 2018;13(4):291–304.	Not RCT

Orang T, Ayoughi S, Moran J, Ghaffari H, Mostafavi S, Rasoulian M, et al. The efficacy of narrative exposure therapy in a sample of Iranian women exposed to ongoing intimate partner violence-A randomized controlled trial. 2018;25(6):827-841.	Wrong age group
Osborn T.L., Rodriguez M., Wasil A.R., Venturo-Conerly K.E., Gan J., Alemu R.G., et al. Single-Session digital intervention for adolescent depression, anxiety, and well-being: Outcomes of a randomized controlled trial with Kenyan adolescents. J Consult Clin Psychol. 2020;88(7):657–68.	No mental health condition
Osborn T, Wasil A. Online Wellness Activities for Kenyan Students. 2020; Available from: https://www.cochranelibrary.com/central/doi/10.1002/central/CN-02239821/full	No mental health condition
Özyurt G, Gencer Ö, Öztürk Y, Özbek A. Is Triple P effective in childhood anxiety disorder? A randomized controlled study. Psychiatry Clin Psychopharmacol. 2019 Oct 2;29(4):570–8.	Wrong age group
Pakpour A.H., Fazeli S., Zeidi I.M., Alimoradi Z., Georgsson M., Brostrom A., et al. Effectiveness of a mobile app-based educational intervention to treat internet gaming disorder among Iranian adolescents: study protocol for a randomized controlled trial. Trials. 2022;23(1):229.	Not a group intervention
Parikh R, Michelson D, Malik K, Shinde S, Weiss HA, Hoogendoorn A, et al. The effectiveness of a low-intensity problem-solving intervention for common adolescent mental health problems in New Delhi, India: protocol for a school-based, individually randomized controlled trial with an embedded stepped-wedge, cluster randomized controlled recruitment trial. Trials. 2019 Dec;20(1):568.	Protocol only
Pegado P., Alckmin-Carvalho F., Leme D., Carneiro F., Kypriotis P., Camacho P., et al. Development, applicability and effects of a pilot program of group cognitive-behavioral therapy in Brazilian adolescents with anorexia nervosa. Rev Psiquiatr Clin. 2018;45(3):57–60.	Not RCT
Peltonen K, Kangaslampi S. Treating children and adolescents with multiple traumas: a randomized clinical trial of narrative exposure therapy. Eur J Psychotraumatology. 2019;10(1):1558708.	Not LMIC
Petersen I, Hanass Hancock J, Bhana A, Govender K. A group-based counselling intervention for depression comorbid with HIV/AIDS using a task shifting approach in South Africa: a randomized controlled pilot study. 2014;158:78-84.	Wrong age group
Pheh KS, Tan KA, Ibrahim N, Sidik S. Effectiveness of online mindfulness-based intervention (Imbi) on inattention, hyperactivity–impulsivity, and executive functioning in college emerging adults with attention-deficit/hyperactivity disorder: a study protocol. 2021;18(3):1-12.	Wrong age group
Pluess M. Phone-Delivered Psychological Intervention (t-CETA) for Mental Health Problems in 8-16 Year-Old Syrian Refugee Children. 2019; Available from: https://www.cochranelibrary.com/central/doi/10.1002/central/CN-01919367/full	Not a group intervention
Polanczyk GV. Brief Internet-delivered Intervention for Children and Adolescents With Anxiety and Depression Symptoms. 2021; Available from: https://www.cochranelibrary.com/central/doi/10.1002/central/CN-02353350/full	Protocol only
Popolo R, MacBeth A, Canfora F, Rebecchi D, Toselli C, Salvatore G, et al. Metacognitive Interpersonal Therapy in group (MIT-G) for young adults with personality disorders: A pilot randomized controlled trial. Psychol Psychother Theory Res Pract. 2019;92(3):342–58.	Wrong age group
Qouta S.R., Palosaari E., Diab M., Punamaki R.L. Intervention effectiveness among war-affected children: a cluster randomized controlled trial on improving mental health. J Trauma Stress. 2012;25(3):288–98.	No mental health condition
Rahman A. Early Adolescent Skills for Emotions (EASE)-Pilot Cluster Randomized Controlled Trial (cRCT) in Public Schools of Rural Pakistan. 2020; Available from: https://www.cochranelibrary.com/central/doi/10.1002/central/CN-02072148/full	Protocol only
Rahman A, Wissow L. SCHOOL HEALTH IMPLEMENTATION NETWORK: EASTERN MEDITERRANEAN REGION. 2019; Available from: https://www.cochranelibrary.com/central/doi/10.1002/central/CN-01984297/full	Protocol only

Raj MA, Kumaraiah V, Bhide A. Cognitive-behavioural intervention in deliberate self-harm. 2001;104(5):340-345.	Wrong age group
Reaven J. Facing Your Fears in Schools: implementing a CBT Program for Students With ASD or Other Special Learning Needs. 2018; Available from: https://www.cochranelibrary.com/central/doi/10.1002/central/CN-01706504/full	Not LMIC
Ren Z, Li X, Zhao L, Yu X, Li Z, Lai L, et al. Effectiveness and mechanism of internet-based self-help intervention for depression: The Chinese version of MoodGYM. Acta Psychol Sin. 2016;48(7):818–32.	Not a group intervention
Richards D. Internet delivered treatment for depression in Colombia. 2012; Available from: https://www.cochranelibrary.com/central/doi/10.1002/central/CN-01860349/full	Wrong age group
Richards J. A sport-for-development intervention for the physical and mental health of young adolescents in Gulu, Uganda. 2012; Available from: https://www.cochranelibrary.com/central/doi/10.1002/central/CN-01873684/full	No psychosocial intervention
Robjant K, Koebach A, Schmitt S, Chibashimba A, Carleial S, Elbert T. The treatment of posttraumatic stress symptoms and aggression in female former child soldiers using adapted Narrative Exposure therapy – a RCT in Eastern Democratic Republic of Congo. Behav Res Ther. 2019 Dec;123:103482.	Not a group intervention
Rodriguez MA. The use of task-sharing to improve treatment engagement in an online mindfulness intervention for stress among Chinese college students. Diss Abstr Int Sect B Sci Eng. 2019;80(2-B(E)):No-Specified.	Wrong age group
Rojas G., Fritsch R., Solis J., Jadresic E., Castillo C., Gonzalez M., et al. Treatment of postnatal depression in low-income mothers in primary-care clinics in Santiago, Chile: a randomised controlled trial. Lancet. 2007;370(9599):1629–37.	Wrong age group
Rosner R., Konig H.-H., Neuner F., Schmidt U., Steil R. Developmentally adapted cognitive processing therapy for adolescents and young adults with PTSD symptoms after physical and sexual abuse: Study protocol for a randomized controlled trial. Trials. 2014;15(1):195.	Not LMIC
Rossello J., Bernal G., Rivera-Medina C. Individual and Group CBT and IPT for Puerto Rican Adolescents With Depressive Symptoms. Cultur Divers Ethnic Minor Psychol. 2008;14(3):234–45.	Not LMIC
Rossello J, Bernal G. The Efficacy of Cognitive-Behavioral and Interpersonal Treatments for Depression in Puerto Rican Adolescents.	Not LMIC
Rossouw J, Yadin E, Alexander D, Seedat S. Long-term follow-up of a randomised controlled trial of prolonged exposure therapy and supportive counselling for post-traumatic stress disorder in adolescents: a task-shifted intervention. Psychol Med. 2022 Apr;52(6):1022–30.	Not a group intervention
Safaralinezhad A, Oveisi S, Sarichlu M, Jourabchi Z. Effect of cognitive-behavioral group therapy on gestational depression: a clinical trial. 2018;21(2):48-59.	Wrong outcomes
Salamanca-Sanabria A, Richards D, Timulak L, Connell S, Perilla M, Parra-Villa Y, et al. A culturally adapted cognitive behavioral internet-delivered intervention for depressive symptoms: randomized controlled trial. 2020;7(1). Available from: https://www.cochranelibrary.com/central/doi/10.1002/central/CN-02214294/full	Wrong age group
Salamanca-Sanabria A., Richards D., Timulak L., Castro-Camacho L., Mojica-Perilla M., Parra-Villa Y. Assessing the efficacy of a culturally adapted cognitive behavioural internet-delivered treatment for depression: Protocol for a randomised controlled trial. BMC Psychiatry. 2018;18(1):53.	Wrong age group
Salmanian M, Ghobari-Bonab B, Hooshyari Z, Mohammadi MR. Effectiveness of spiritual psychotherapy on attachment to God among adolescents with conduct disorder: A randomized controlled trial. Psychol Relig Spiritual. 2020;12(3):269–75.	Wrong outcomes
Samantaray N, Nath B, Behera N, Mishra A, Singh P, Sudhir P. Brief cognitive behavior group therapy for social anxiety among medical students: a randomized placebo-controlled trial. 2021;55:102526.	Wrong age group

Santamarina Perez P., Romero Cela S., Mendez Blanco I., Font Martinez E., Picado Rossi M., Martinez Mallen E., et al. Efficacy of dialectical behavior therapy compared to supportive therapy in adolescents with suicidal behavior. Eur Neuropsychopharmacol. 2017;27(Supplement 4):S853–4.	Not LMIC
Santamarina-Perez P, Mendez I, Singh M, Berk M, Picado M, Font E, et al. Adapted Dialectical Behavior Therapy for Adolescents with a High Risk of Suicide in a Community Clinic: a Pragmatic Randomized Controlled Trial. 2020;50(3):652-667.	Not LMIC
Schaal S, Elbert T, Neuner F. Narrative exposure therapy versus interpersonal psychotherapy. A pilot randomized controlled trial with Rwandan genocide orphans. 2009;78(5):298-306.	Wrong age group
Schaal S. Narrative Exposure Therapy Versus Group Interpersonal Psychotherapy. 2008; Available from: https://www.cochranelibrary.com/central/doi/10.1002/central/CN-01517220/full	Wrong age group
Schauer E. Trauma Treatment for Children in War : build-up of an evidence-based large-scale mental health intervention in North-Eastern Sri Lanka.	Not a group intervention
Seghati T, Shafiabady A, Soodani M, Jofreh MG. Effectiveness of logotheraphy and cognitive-behavioral therapy on life expectancy among students with depression disorder: a clinical trial study. J Gorgan Univ Med Sci. 2021;23(2):fa24–32.	Wrong age group
Shabani MJ, Mohsenabadi H, Omidi A, Lee EB, Twohig MP, Ahmadvand A, et al. An Iranian study of group acceptance and commitment therapy versus group cognitive behavioral therapy for adolescents with obsessive-compulsive disorder on an optimal dose of selective serotonin reuptake inhibitors. J Obsessive-Compuls Relat Disord. 2019 Jul 1;22:100440.	Wrong diagnosis
Shannon C. Psychological and Psychosocial Intervention With War-Affected Children. 2012; Available from: https://www.cochranelibrary.com/central/doi/10.1002/central/CN-01534909/full	Not RCT
Sharma P, Mehta M, Sagar R. Efficacy of transdiagnostic cognitive-behavioral group therapy for anxiety disorders and headache in adolescents. J Anxiety Disord. 2017 Mar;46:78–84.	Clinical setting
Shein-Szydlo J, Sukhodolsky DG, Kon DS, Tejeda MM, Ramirez E, Ruchkin V. A Randomized Controlled Study of Cognitive-Behavioral Therapy for Posttraumatic Stress in Street Children in Mexico City. J Trauma Stress. 2016 Oct;29(5):406–14.	Not a group intervention
Shi J, Wang L, Yao Y, Zhan C, Su N, Zhao X. Systemic Therapy for Youth at Clinical High Risk for Psychosis: A Pilot Study. Front Psychiatry. 2017 Oct 20;8:211.	Wrong diagnosis
Simms V, Weiss HA, Chinoda S, Mutsinze A, Bernays S, Verhey R, et al. Peer-led counselling with problem discussion therapy for adolescents living with HIV in Zimbabwe: A cluster-randomised trial. Bor J, editor. PLOS Med. 2022 Jan 5;19(1):e1003887.	No mental health condition
Simsek MK, Secer I. Developing and Examining the Effectiveness of a Cognitive Behavioral Therapy-Based Psychoeducation Practice for Reducing Obsessive-Compulsive Symptoms in Adolescents: A Mixed-Methods Study With a Turkish Sample. Front Psychol. 2022;13(101550902):805035.	Wrong age group
So C, Leung P, Hung S. Treatment effectiveness of combined medication/behavioural treatment with chinese ADHD children in routine practice. 2008;46(9):983-992.	Not LMIC
So YC. Effectiveness of methylphenidate and combined treatment (methylphenidate and psychosocial treatment) for Chinese children with attention-deficit/hyperactivity disorder in a community mental health center. Diss Abstr Int Sect B Sci Eng. 2007;67(7-B):4119.	Not LMIC
Soares EE. Analysis of suicide risk factors after use of an online depression prevention program (CATCH-IT) for adolescents. Diss Abstr Int Sect B Sci Eng. 2022;83(5-B):No-Specified.	Not LMIC
Srivastava P, Mehta M, Sagar R, Ambekar A. Smartteen- a computer assisted cognitive behavior therapy for Indian adolescents with depression- a pilot study. Asian J Psychiatry. 2020 Apr;50:101970.	Not a group intervention

Sumathipala A, Siribaddana S, Abeysingha M, De Silva P, Dewey M, Prince M, et al. Cognitive-behavioural therapy v. structured care for medically unexplained symptoms: randomised controlled trial. 2008;193(1):51-59.	Wrong outcomes
Sunetr B, Puangpaka K, Quinn Griffin M. Effectiveness of an Early Depression Prevention Program on Coping Skills and Depression among Pregnant Adolescents: a Randomized Controlled Trial. 2022;26(2):296-312.	No mental health condition
Tajrishi M, Abbasi S, Fard T, Yousefi S, Abadi A, Kasmaei H. Efficacy of attribution retraining on mental health of epileptic children. 2015;17(10) (no pagination). Available from: https://www.cochranelibrary.com/central/doi/10.1002/central/CN-01131998/full	Not RCT
Tang X, Wong DFK. Evaluation of a Prevention Program for Depression among High School Adolescent in Mainland China: a Cluster Randomized Controlled Trial. 2019; Available from: https://www.cochranelibrary.com/central/doi/10.1002/central/CN-01974392/full	No mental health condition
Thurman T.R., Luckett B.G., Nice J., Spyrelis A., Taylor T.M. Effect of a bereavement support group on female adolescents' psychological health: a randomised controlled trial in South Africa. Lancet Glob Health. 2017;5(6):e604–14.	No mental health condition
Thurman T.R., Nice J., Taylor T.M., Luckett B. Mitigating depression among orphaned and vulnerable adolescents: a randomized controlled trial of interpersonal psychotherapy for groups in South Africa. Child Adolesc Ment Health. 2017;22(4):224–31.	No mental health condition
Tran T., Nguyen H.T., Shochet I., Wurfl A., Orr J., Nguyen N., et al. School-based, two-arm, parallel, controlled trial of a culturally adapted resilience intervention to improve adolescent mental health in Vietnam: Study protocol. BMJ Open. 2020;10(10):e039343.	No mental health condition
Tulbure B, Andersson G, Sălăgean N, Pearce M, Koenig H. Religious versus Conventional Internet-based Cognitive Behavioral Therapy for Depression. 2018;57(5):1634-1648.	Wrong age group
Ugwuanyi C.S., Ede M.O., Onyishi C.N., Ossai O.V., Nwokenna E.N., Obikwelu L.C., et al. Effect of cognitive-behavioral therapy with music therapy in reducing physics test anxiety among students as measured by generalized test anxiety scale. Medicine (Baltimore). 2020;99(17):e16406.	No mental health condition
University of Botswana. The Effectiveness of CBI on Depression and Adherence in HIV Infected Adolescents. 2022; Available from: https://www.cochranelibrary.com/central/doi/10.1002/central/CN-02431470/full	Clinical setting
University of Pennsylvania. Evaluating an Online Wellness Intervention for Indian College Students. 2020; Available from: https://www.cochranelibrary.com/central/doi/10.1002/central/CN-02182234/full	Wrong age group
Vagos P. Changing the Course of Social Anxiety in Adolescence. 2021; Available from: https://www.cochranelibrary.com/central/doi/10.1002/central/CN-02297064/full	Not LMIC
Van De Water T, Rossouw J, Van Der Watt ASJ, Yadin E, Seedat S. Adolescents' Experience of Stigma When Accessing School-Based PTSD Interventions. Qual Health Res. 2018 Jun;28(7):1088–98.	Wrong outcomes
Van De Water T, Rossouw J, Yadin E, Seedat S. Impediments and catalysts to task-shifting psychotherapeutic interventions for adolescents with PTSD: perspectives of multi-stakeholders. Child Adolesc Psychiatry Ment Health. 2017 Dec;11(1):48.	Not a group intervention
Van De Water T, Rossouw J, Yadin E, Seedat S. Adolescent and nurse perspectives of psychotherapeutic interventions for PTSD delivered through task-shifting in a low resource setting. Yotebieng M, editor. PLOS ONE. 2018 Jul 10;13(7):e0199816.	Not a group intervention
Venkatesan P. The BASIC group: closing mental health-care gaps for children. Lancet Psychiatry. 2020;7(2):130–1.	Not RCT
Visagie L, Loxton H, Silverman WK. Research Protocol: Development, implementation and evaluation of a cognitive behavioural therapy-based intervention programme for the management of anxiety symptoms in South African children with visual impairments. Afr J Disabil. 2015;4(1):160.	No mental health condition

Visagie L, Loxton H, Swartz L, Stallard P. Cognitive behaviour therapy-based early intervention and prevention programme for anxiety in South African children with visual impairments. Afr J Disabil. 2021;10(101623460):796.	No mental health condition
Waldemar J.O.C., Rigatti R., Menezes C.B., Guimaraes G., Falceto O., Heldt E. Impact of a combined mindfulness and social-emotional learning program on fifth graders in a Brazilian public school setting. Psychol Neurosci. 2016;9(1):79–90.	Not RCT
Wang DC, Aten JD, Boan D, Jean-Charles W, Griff KP, Valcin VC, et al. Culturally adapted spiritually oriented trauma-focused cognitive-behavioral therapy for child survivors of restavek. Spiritual Clin Pract. 2016;3(4):224–36.	Not RCT
Wang L., Guo Y., Liu Y., Yan X., Ding R. The effects of a mobile phone-based psychological intervention program on stress, anxiety and self-efficacy among undergraduate nursing students during clinical practice: A randomized controlled trial. J Prof Nurs Off J Am Assoc Coll Nurs. 2022;42((Wang) Nursing Department of Affiliated Hospital of Zunyi Medical University, ZunYi 563000, Guizhou, China; Nursing College of Zunyi Medical University, Zunyi 563000, Guizhou, China. Electronic address: 2726912415@qq.com(Guo, Liu, Yan, Ding) Nursing Colle):219–24.	Wrong age group
Wasil A.R., Osborn T.L., Weisz J.R., Derubeis R.J. Online single-session interventions for Kenyan adolescents: Study protocol for a comparative effectiveness randomised controlled trial. Gen Psychiatry. 2021;34(3):e100446.	No mental health condition
Wong A, Ting K, Chen E. Group cognitive behavioural therapy for Chinese patients with psychotic disorder: a feasibility controlled study. 2019;39:157-164.	Wrong age group
Xu Y, Wu T, Yu Y, Li M. A randomized controlled trial of well-being therapy to promote adaptation and alleviate emotional distress among medical freshmen. 2019;19(1):182.	Wrong age group
Yang J., Lei D., Suo X., Tallman M.J., Qin K., Li W., et al. A preliminary study of the effects of mindfulness-based cognitive therapy on structural brain networks in mood-dysregulated youth with a familial risk for bipolar disorder. Early Interv Psychiatry. 2022;16(9):1011–9.	Wrong outcomes
Yang X., Liu D., Wang Y., Chen Y., Chen W., Yang C., et al. Effectiveness of Zhong-Yong thinking based dialectical behavior therapy group skills training versus supportive group therapy for lowering suicidal risks in Chinese young adults: A randomized controlled trial with a 6-month follow-up. Brain Behav. 2020;10(6):e01621.	Wrong age group
Yang X, Zhao J, Chen Y, Zu S, Zhao J. Comprehensive self-control training benefits depressed college students: A six-month randomized controlled intervention trial. J Affect Disord. 2018 Jan;226:251–60.	Wrong age group
Yator O, Khasakhala L, John-Stewart G, Kumar M. Acceptability and Feasibility of Group Interpersonal Therapy (IPT-G) for Depressed HIV+ Postpartum Adolescents Delivered by Community Health Workers: a Protocol Paper. 2020;11. Available from: https://www.cochranelibrary.com/central/doi/10.1002/central/CN-02207867/full	Wrong age group
Yoo H.-J., Bahn G., Cho I.-H., Kim E.-K., Kim J.-H., Min J.-W., et al. A randomized controlled trial of the korean version of the PEERS parent-assisted social skills training program for teens with ASD. Autism Res. 2014;7(1):145–61.	Not LMIC
Zarghami F, Heydarinasab L, Shairi MR, Shahrivar Z. The effectiveness of cognitive behavior treatment based on Kendall's Coping Program on anxiety disorders: A transdiagnostic approach. Dev Psychol J Iran Psychol. 2015;12(45):37–50.	Not RCT
Zeng H, Liu S, Huang R, Zhou Y, Tang J, Xie J, et al. Effect of the TEACCH program on the rehabilitation of preschool children with autistic spectrum disorder: A randomized controlled trial. J Psychiatr Res. 2021 Jun;138:420–7.	Wrong diagnosis
ZHANG DD, WONG SY. The effects of a mindfulness based intervention - MYmind - for children with ADHD and their parents: a randomised controlled trial. 2018; Available from: https://www.cochranelibrary.com/central/doi/10.1002/central/CN-01899562/full	Wrong age group

Zhang HJ, Dong XL, Zhang YF, Fang YF, Zhang HY. [Effect of combination of acupuncture and psychological intervention on attention, response inhibition and cerebral blood flow in children with attention deficit hyperactivity disorder]. Zhongguo Zhen Jiu Chin Acupunct Moxibustion. 2021;41(4):400–4.	No psychosocial intervention
Zhang Y., Huang K., Cong H., Wang M. EFFECTS OF GROUP PSYCHOLOGICAL COUNSELING ON NONSUICIDAL SELF-INJURY (NSSI) BEHAVIORS OF COLLEGE STUDENTS WITH DEPRESSION. Psychiatr Danub. 2022;34(2):229–35.	Wrong age group
Zhao Y., Munro-Kramer M.L., Shi S., Wang J., Luo J. A randomized controlled trial: effects of a prenatal depression intervention on perinatal outcomes among Chinese high-risk pregnant women with medically defined complications. Arch Womens Ment Health. 2017;20(2):333–44.	Wrong age group
Zhu Z, Wang R, Kao H, Zong Y, Liu Z, Tang S, et al. Effect of calligraphy training on hyperarousal symptoms for childhood survivors of the 2008 china earthquakes. 2014;10:977-984.	No mental health condition
Zu S, Xiang Y, Liu J, Zhang L, Wang G, Ma X, et al. A comparison of cognitive-behavioral therapy, antidepressants, their combination and standard treatment for Chinese patients with moderate-severe major depressive disorders. 2014;152-154:262-267.	Wrong age group
Ahmad Othman A, Wan Jaafar WM, Zainudin ZN, Yusop YM. Effectiveness of cognitive behavior therapy and acceptance and commitment therapy on depression and anxiety among emerging adults in malaysia. Curr Psychol J Diverse Perspect Diverse Psychol Issues. 2023;No-Specified.	Wrong age group
Ahmadi E, Hatami SM, Hashemi-Razni H, Sepahmansour M. Reducing anxiety in cancer patients using mindfulness-based stress reduction and transcranial direct current stimulation: an intervention for cancer. Chronic Dis J. 2023;11(3):153–9.	Not RCT
Aneke AO, Ede MO, Agbigwe IB, Obumse NA, Nnamani O, Ngwoke AN, et al. Examining the impact of randomized control intervention on depressive symptoms in schoolchildren with atypical behaviors. Medicine (Baltimore). 2023;102(7):e32964.	Wrong outcomes
Anwuri A. Management of psychosocial problems of adolescents in some orphanages in south-west Nigeria. Diss Abstr Int Sect B Sci Eng. 2023;84(2):No-Specified.	Not RCT
Ardi Z, Eseadi C, Yuniarti E, Yendi FM, Murni AW. Efficacy of Cognitive Behavioral Therapy With Local Wisdom and Web-Based Counseling on Generalized Anxiety Disorders and Functional Gastrointestinal Disorders in Adolescent College Girls: protocol for a Randomized Controlled Trial. JMIR Res Protoc. 2023;12:e50316.	Wrong diagnosis
Bosqui T, McEwen FS, Chehade N, Moghames P, Skavenski S, Murray L, et al. What drives change in children receiving telephone-delivered Common Elements Treatment Approach (t-CETA)? A multiple n = 1 study with Syrian refugee children and adolescents in Lebanon. Child Abuse Negl. 2023;106388.	Not a group intervention
Bryant RA, Bawaneh A, Awwad M, Al-Hayek H, Giardinelli L, Whitney C, et al. Twelve-month follow-up of a randomised clinical trial of a brief group psychological intervention for common mental disorders in Syrian refugees in Jordan. Epidemiol Psychiatr Sci [Internet]. 2022;31. Available from: http://ovidsp.ovid.com/ovidweb.cgi?T=JS&PAGE=reference&D=psyc22&NEWS=N&AN=2023-20166-001	Wrong age group
Byansi W, Ssewamala FM, Neilands TB, Bahar OS, Nabunya P, Namuwonge F, et al. The short-term impact of a combination intervention on depressive symptoms among school-going adolescent girls in southwestern Uganda: the Suubi4Her cluster randomized trial. J Adolesc Health. 2022;71(3):301–7.	Not a treatment intervention
Demir S, Ercan F. The effectiveness of cognitive behavioral therapy-based group counseling on depressive symptomatology, anxiety levels, automatic thoughts, and coping ways Turkish nursing students: A randomized controlled trial. Perspect Psychiatr Care. 2022;58(4):2394–406.	Wrong age
Desrosiers A, Carrol B, Ritsema H, Higgins W, Momoh F, Betancourt TS. Advancing sustainable implementation of an evidence-based mental health intervention in Sierra Leone's schools: protocol for a hybrid type 3 implementation-effectiveness trial. BMC Public Health [Internet]. 2024;24(362). Available from: https://link.springer.com/article/10.1186/s12889-024-17928-w	Protocol only

Desrosiers A, Freeman J, Mitra R, Bond L, Santo L dal, Farrar J, et al. Alternative delivery platforms for expanding evidence-based mental health interventions for youth in Sierra Leone: a pilot study. Vulnerable Child Youth Stud. 2023;18(1):131–42.	Wrong age group
Donenberg GR, Fitts J, Ingabire C, Nsanzimana S, Fabri M, Emerson E, et al. Results of the kigali imbereheza project: a 2-arm individually randomized trial of TI-CBT enhanced to address Art adherence and mental health for Rwandan youth living with HIV. JAIDS J Acquir Immune Defic Syndr. 2022;90(1):69–78.	Not a treatment intervention
Dozio E. Emotional stabilization interventions for people exposed to chronic traumatic events, in humanitarian settings. Eur Psychiatry. 2023;66:S178.	Wrong outcomes
Ediz C, Budak FK. Effects of psychosocial support-based psychoeducation for Turkish pregnant adolescents on anxiety, depression and perceived social support: a randomized controlled study. Rural Remote Health [Internet]. 2023;23(3). Available from: https://www.rrh.org.au/journal/article/7553	Not a treatment intervention
Friberg PA. NutriMind: a Combination of Healthy Diet and Psychotherapy to Treat Depression. https://clinicaltrials.gov/show/NCT05848973 [Internet]. 2023; Available from: https://www.cochranelibrary.com/central/doi/10.1002/central/CN-02559984/full	Wrong age group
Gautam K. School-based IPT-G for Adolescents With Depression in Nepal: a Pilot Realist C-RCT. https://clinicaltrials.gov/ct2/show/NCT06017700 [Internet]. 2023; Available from: https://www.cochranelibrary.com/central/doi/10.1002/central/CN-02594386/full	Protocol only
Ghazal L. Reducing Depression and Anxiety Among Teens. https://clinicaltrials.gov/ct2/show/NCT06155838 [Internet]. 2023; Available from: https://www.cochranelibrary.com/central/doi/10.1002/central/CN-02632363/full	Not a treatment intervention
Guo J. Effectiveness of School-Based Psychosocial Interventions on Mental Health Among Chinese Rural Children with Traumatic Experiences: a Cluster Randomized Controlled Trial. https://trialsearch.who.int/Trial2.aspx?TrialID=ChiCTR2300069405 [Internet]. 2023; Available from: https://www.cochranelibrary.com/central/doi/10.1002/central/CN-02565525/full	Not a treatment intervention
Hamdani SU, Huma ZE, Malik A, Nizami AT, Baneen UU, Suleman N, et al. Improving psychosocial distress for young adolescents in rural schools of Pakistan: study protocol of a cluster randomised controlled trial. BMJ Open. 2022;12(9):e063607.	Protocol only
Hanif T. Effectiveness of Child-Parent Relationship Therapy (CPRT). https://clinicaltrials.gov/ct2/show/NCT06087302 [Internet]. 2023; Available from: https://www.cochranelibrary.com/central/doi/10.1002/central/CN-02606203/full	Wrong age group
Jia Q, Wang H, Sun D. Exploring the effect of psychological interventions on anxiety and depression in adolescents based on behavior change wheel theory. Iran J Public Health. 2024;53(2):404–13.	Not a group intervention
Jiang Y, Li X, Harrison SE, Zhang J, Qiao S, Zhao J, et al. Effects of a Multilevel Resilience-Based Intervention on Mental Health for Children Affected by Parental HIV: a Cluster Randomized Controlled Trial. J Child Fam Stud. 2022;31(4):1094-1105.	Not a treatment intervention
Jordans MJD, Bleile ACE. Evaluating the effectiveness of the psychosocial, movement-based intervention TeamUp in Burundi. https://trialsearch.who.int/Trial2.aspx?TrialID=ISRCTN17499603 [Internet]. 2023; Available from: https://www.cochranelibrary.com/central/doi/10.1002/central/CN-02607834/full	Not a treatment intervention
Kacmarek CN, Johnson NE, Osborn TL, Wasanga C, Weisz JR, Yates BT. Costs and cost-effectiveness of Shamiri, a brief, layperson-delivered intervention for Kenyan adolescents: a randomized controlled trial. BMC Health Serv Res. 2023;23(1):827.	Wrong outcomes
Kaminer D, Simmons C, Seedat S, Skavenski S, Murray L, Kidd M, et al. Effectiveness of abbreviated trauma-focused cognitive behavioural therapy for South African adolescents: a randomized controlled trial. Eur J Psychotraumatology. 2023;14(1):2181602.	Not a group intervention
Komariah M, Ibrahim K, Pahria T, Rahayuwati L, Somantri I. Effect of Mindfulness Breathing Meditation on Depression, Anxiety, and Stress: A Randomized Controlled Trial among University Students. Healthc Basel	Wrong age group

Switz [Internet]. 2022;11(1). Available from: https://www.cochranelibrary.com/central/doi/10.1002/central/CN-02550566/full	
Liu S, Li G. Analysis of the Effect of Music Therapy Interventions on College Students with Excessive Anxiety. Occup Ther Int. 2023;2023:3351918.	Wrong age group
Long K. INTERVENTION OF COLLEGE MUSIC EDUCATION INTEGRATING CHINESE TRADITIONAL MUSIC ON STUDENTS' ANXIETY DISORDER. Psychiatr Danub. 2021;33:S88-S90.	Wrong age group
Miller-Graff LE, Cummings EM. Supporting youth and families in Gaza: a randomized controlled trial of a family-based intervention program. Int J Environ Res Public Health [Internet]. 2022;19(14). Available from: https://www.mdpi.com/1660-4601/19/14/8337	Not a treatment intervention
Nabunya P, Ssewamala FM, Kizito S, Mugisha J, Brathwaite R, Neilands TB, et al. Preliminary Impact of Group-Based Interventions on Stigma, Mental Health and Treatment Adherence Among Adolescents Living with HIV in Uganda. J Pediatr. 2024;113983.	Not a treatment intervention
Negash A, Khan MA, Medhin G, Wondimagegn D, Pain C, Araya M. Feasibility and acceptability of brief individual interpersonal psychotherapy among university students with mental distress in Ethiopia. BMC Psychol [Internet]. 2021;9. Available from: http://ovidsp.ovid.com/ovidweb.cgi?T=JS&PAGE=reference&D=psyc22&NEWS=N&AN=2021-41569-001	Not RCT
Neufeld CB, Palma PC, Caetano KAS, Brust-Renck PG, Curtiss J, Hofmann SG. A randomized clinical trial of group and individual Cognitive-Behavioral Therapy approaches for Social Anxiety Disorder. Int J Clin Health Psychol. 2020;20(1):29-37.	Wrong age group
Nezafat Ferizi J, Ashouri A, Gharraee B, Farid AAA. Comparison of the Effectiveness of Interpersonal Counseling and Interpersonal Psychotherapy in Emotional Expression, Social Skills, and Depression Symptoms in Students. Iran J Psychiatry Behav Sci [Internet]. 2023;17(2). Available from: https://www.cochranelibrary.com/central/doi/10.1002/central/CN-02582294/full	Wrong age group
Obiweluozo PE, Ede MO, Onwurah CN, Uzodinma UE, Dike IC, Ejiofor JN, et al. Impact of cognitive behavioural play therapy on social anxiety among school children with stuttering deficit: A cluster randomised trial with three months follow-up. Med U S. 2021;100(19):E24350.	Wrong outcomes
Ochuku B, Osborn TL, Nerima D, van der Markt A, Rusch T, Omune H, et al. Testing pathways to scale: study protocol for a three-arm randomized controlled trial of a centralized and a decentralized ('Train the Trainers') dissemination of a mental health program for Kenyan adolescents. Trials. 2023;24(1):526.	Not a treatment intervention
Odewale O. Effects of dialectical behaviour therapy and social skills training on psycho-social problems of children from single-parent homes in Ibadan, Nigeria. Diss Abstr Int Sect B Sci Eng. 2023;84(2):No-Specified.	Not RCT
Olashore AA, Paruk S, Ogunwale A, Ita M, Tomita A, Chiliza B. The effectiveness of psychoeducation and problem-solving on depression and treatment adherence in adolescents living with HIV in Botswana: an exploratory clinical trial. Child Adolesc Psychiatry Ment Health [Internet]. 2023;17(1). Available from: https://www.cochranelibrary.com/central/doi/10.1002/central/CN-02513864/full	Clinical setting
Osborn TL, Ndetei DM, Sacco PL, Mutiso V, Sommer D. An arts-literacy intervention for adolescent depression and anxiety symptoms: outcomes of a randomised controlled trial of Pre-Texts with Kenyan adolescents. EClinicalMedicine. 2023;66:102288.	Not a treatment intervention
Pandey R, Tiwari GK, Rai PK. Understanding the efficacy of self-affirmation intervention for subclinical depression among young adults. J Pract Clin Psychol. 2023;11(1):23–34.	Wrong age group
Simms V, Weiss HA, Chinoda S, Mutsinze A, Bernays S, Verhey R, et al. Peer-led counselling with problem discussion therapy for adolescents living with HIV in Zimbabwe: a cluster-randomised trial. PLoS Med [Internet]. 2022;19(1). Available from: https://journals.plos.org/plosmedicine/article?id=10.1371/journal.pmed.1003887	Clinical setting

Sit HF, Ling R, Lam AIF, Chen W, Latkin CA, Hall BJ. The cultural adaptation of step-by-step: An intervention to address depression among Chinese young adults. Front Psychiatry [Internet]. 2020;11. Available from: http://ovidsp.ovid.com/ovidweb.cgi?T=JS&PAGE=reference&D=psyc19&NEWS=N&AN=2020-54141-001	Not RCT
Soleimani Rad H, Goodarzi H, Bahrami L, Abolghasemi A. Internet-based versus face-to-face cognitive-behavioral therapy for social anxiety disorder: A randomized control trial. Behav Ther. 2023;No-Specified.	Clinical setting
Sorsdahl K, Van der Westhuizen C, Hornsby N, Jacobs Y, Poole M, Neuman M, et al. Project ASPIRE: a feasibility randomized controlled trial of a brief intervention for reducing risk of depression and alcohol-related harms among South African adolescents. Psychother Res. 2024;34(1):96-110.	Wrong outcomes
Ssewamala FM, Brathwaite R, Sensoy Bahar O, Namatovu P, Neilands TB, Kiyingi J, et al. The Post-intervention Impact of Amaka Amasanyufu on Behavioral and Mental Health Functioning of Children and Adolescents in Low-Resource Communities in Uganda: analysis of a Cluster-Randomized Trial From the SMART Africa-Uganda Study (2016-2022). J Adolesc Health. 2023;72(5):S3-S10.	Wrong outcomes
Velloza J. Friendship Bench Mental Health Intervention for Adolescent Girls and Young Women in South African PrEP Delivery Settings. https://clinicaltrials.gov/show/NCT05664490 [Internet]. 2022; Available from: https://www.cochranelibrary.com/central/doi/10.1002/central/CN-02507602/full	No mental health condition
Victor-Aigbodion V, Eseadi C, Ardi Z, Sewagegn AA, Ololo K, Abonor LB, et al. Effectiveness of rational emotive behavior therapy in reducing depression among undergraduate medical students. Medicine (Baltimore). 2023;102(4):e32724.	Wrong age group
Yi C, Naixin L, Iyendo TO, Apuke OD. Cognitive behavior, art, and music therapies intervention for treating the depression of children: A randomized control trial. Psychiatry Res. 2024;333:115716.	Wrong outcomes
Zuo Z, Zhang X. A randomized controlled trial of group CBT with positive psychotherapy intervention for university students with maladaptive perfectionism in China. Front Psychol. 2023;14:1161575.	Wrong age group
