# Supplementary material for: Group psychosocial interventions for anxiety, depression, and post-traumatic stress disorder in children and adolescents in low- and middle-income countries: A realist systematic review and meta-analysis of randomised controlled trials
Source: PLOS Ment Health. 2026 Jan 29;3(1):e0000533. doi: 10.1371/journal.pmen.0000533 (PMC12854475; doi:10.1371/journal.pmen.0000533)
Supplement: S1 Table — (PDF) [file pmen.0000533.s003.pdf]

S1 Table. Risk of bias ratings: Cochrane Risk of Bias 2 tool.

Author, year	Risk of bias arising from the randomization process	Risk of bias due to deviations from the intended interventions	Risk of bias due to missing outcome data	Risk of bias in measurement of the outcome	Risk of bias in selection of the reported result	Overall Risk of Bias
Ahmadi et al., 2022	Low risk	Low risk	Low risk	Low risk	Low risk	Low risk
Akhtar et al., 2021	Low risk	Low risk	Low risk	Low risk	Low risk	Low risk
Barron et al., 2013	Some concerns	Low risk	Low risk	Low risk	Low risk	Some concerns
Barron et al., 2016	Low risk	Low risk	Some concerns	Low risk	Low risk	Some concerns
Barron et al., 2021	Low risk	Low risk	Low risk	Low risk	Low risk	Low risk
Bella-Awusah et al., 2016	Low risk	Low risk	Low risk	Some concerns	Low risk	Some concerns
Betancourt et al., 2012	Low risk	Low risk	Low risk	Low risk	Low risk	Low risk
Betancourt et al., 2014	Low risk	Low risk	Low risk	Low risk	Low risk	Low risk
Bolton et al., 2007	Low risk	Low risk	Low risk	Low risk	Low risk	Low risk
Bryant et al., 2022	Low risk	Low risk	Low risk	Low risk	Low risk	Low risk
Chen et al., 2014	Some concerns	High risk	High risk	Low risk	Low risk	High risk
Daryabeigi et al., 2020	Low risk	Some concerns	Some concerns	Some concerns	Low risk	High risk
Dorsey et al., 2021	Low risk	Low risk	Low risk	Low risk	Low risk	Low risk
Ede et al., 2020	Low risk	Low risk	Low risk	Low risk	Low risk	Low risk
El-Khani et al., 2021	Low risk	Low risk	Low risk	Low risk	Low risk	Low risk
Getanda et al., 2020	Low risk	Low risk	Some concerns	Some concerns	Low risk	Some concerns
Gordon et al., 2008	Low risk	Low risk	Low risk	Some concerns	Low risk	Some concerns
Jacob et al., 2016	Low risk	High risk	High risk	Some concerns	Low risk	High risk
Jordans et al., 2010	Low risk	Low risk	Low risk	Low risk	Low risk	Low risk
Jordans et al., 2023	Low risk	Low risk	High risk	Low risk	Low risk	High risk
Kaesornsamut et al., 2012	Some concerns	Low risk	Low risk	Low risk	Low risk	Some concerns
Kalantari et al., 2012	Low risk	Low risk	Some concerns	Some concerns	Low risk	Some concerns
Khalid et al., 2022	Low risk	Low risk	Some concerns	Low risk	Low risk	Some concerns
Khan et al., 2020	Low risk	Low risk	Some concerns	Some concerns	Low risk	Some concerns
Layne et al., 2008	Low risk	Low risk	High risk	Low risk	Low risk	High risk
Li et al., 2022	Low risk	Low risk	Low risk	Low risk	Low risk	Low risk
Li et al., 2023	Low risk	Low risk	Some concerns	Low risk	Low risk	Some concerns
McMullen et al., 2013	Low risk	Low risk	Low risk	Low risk	Low risk	Low risk
O'Callaghan et al., 2013	Low risk	Low risk	Low risk	Low risk	Low risk	Low risk
Osborn et al., 2021	Low risk	Low risk	Low risk	Low risk	Low risk	Low risk
Peter et al., 2022	Low risk	Low risk	Some concerns	Low risk	Low risk	Some concerns
Pityaratstian et al., 2015	Low risk	Low risk	Low risk	Low risk	Low risk	Low risk
Saw et al., 2019	Low risk	Low risk	Low risk	Low risk	Low risk	Low risk
Saw et al., 2020	Low risk	Low risk	Low risk	Low risk	Low risk	Low risk
Tol et al., 2008	Some concerns	Low risk	Low risk	Some concerns	Low risk	Some concerns
Tol et al., 2012	Low risk	Low risk	Low risk	Low risk	Low risk	Low risk
Tol et al., 2014	Low risk	Low risk	Some concerns	Low risk	Low risk	Some concerns
Ugwu et al., 2022	Low risk	Low risk	Low risk	Some concerns	Low risk	Some concerns
Zafar et al., 2015	Low risk	Low risk	Some concerns	Some concerns	Low risk	Some concerns
