# Supplementary figures and images for: Group psychosocial interventions for anxiety, depression, and post-traumatic stress disorder in children and adolescents in low- and middle-income countries: A realist systematic review and meta-analysis of randomised controlled trials
Source: PLOS Ment Health. 2026 Jan 29;3(1):e0000533. doi: 10.1371/journal.pmen.0000533 (PMC12854475; doi:10.1371/journal.pmen.0000533)

# S1 File. Funnel plots.

## Funnel plots – depression

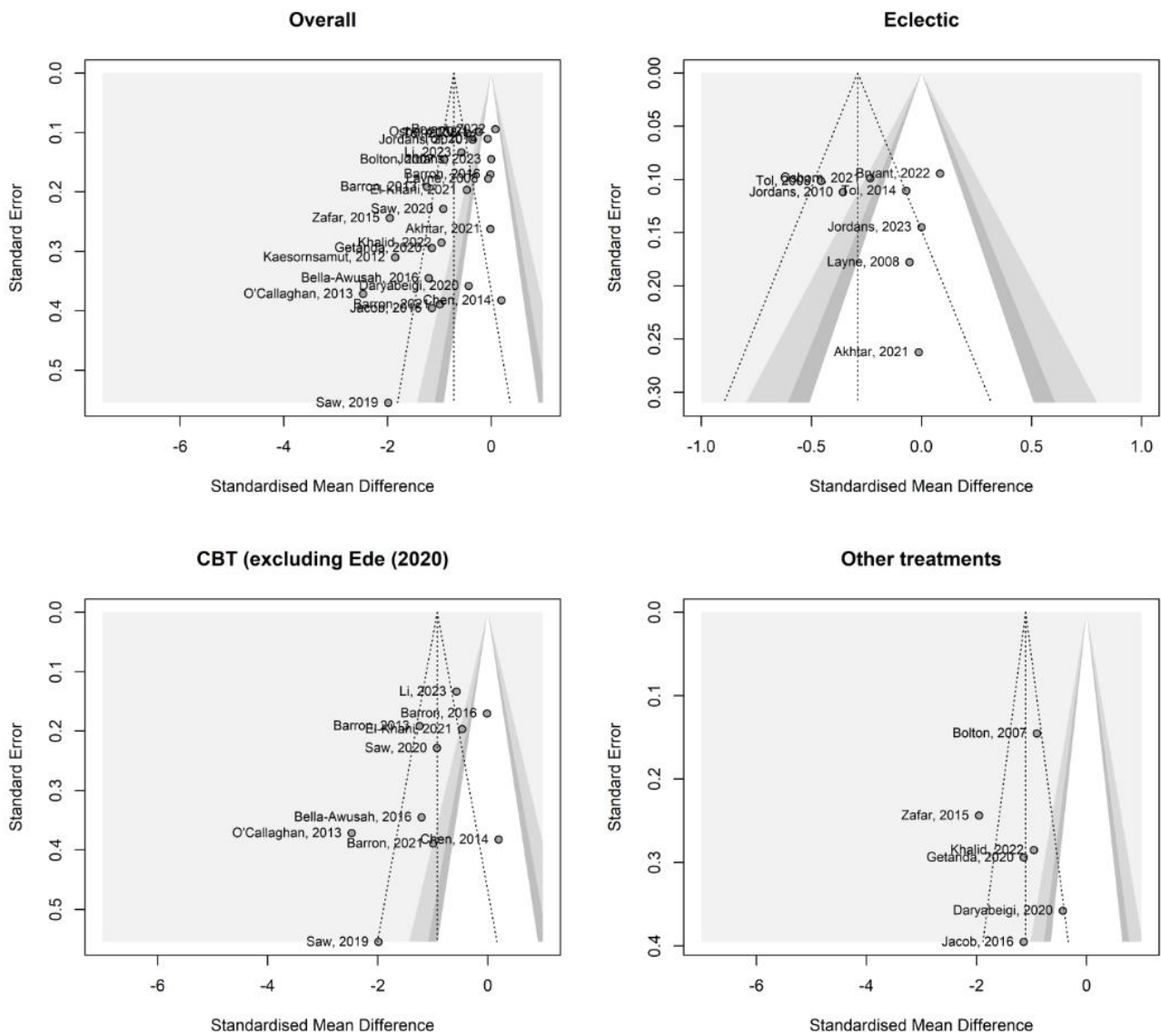

## Funnel plot - anxiety

### Overall

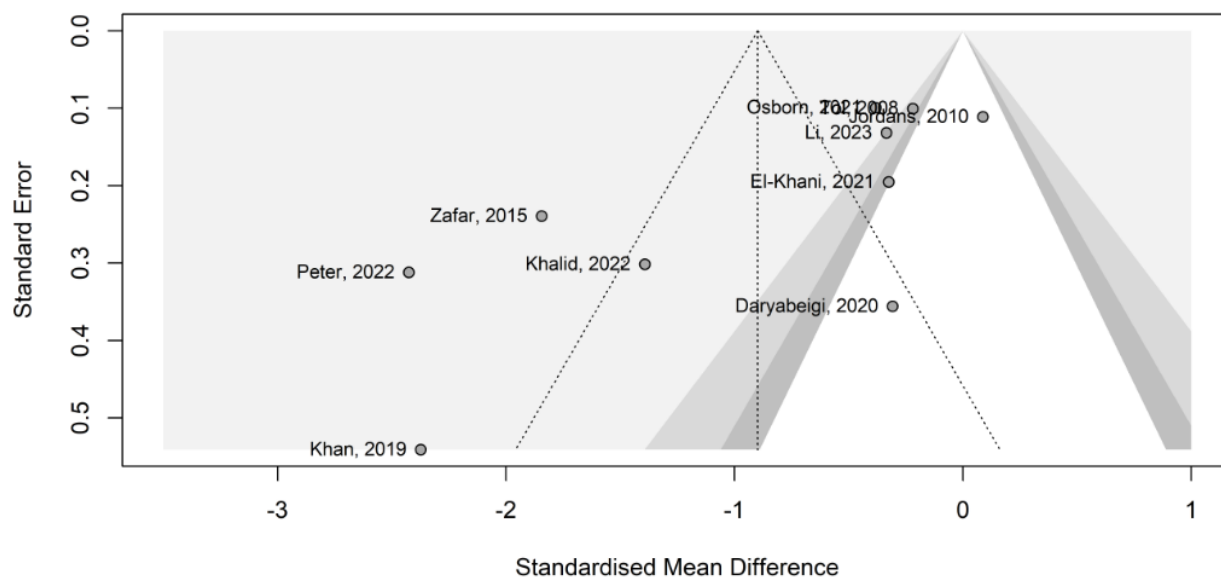

### Funnel plot – PTSD

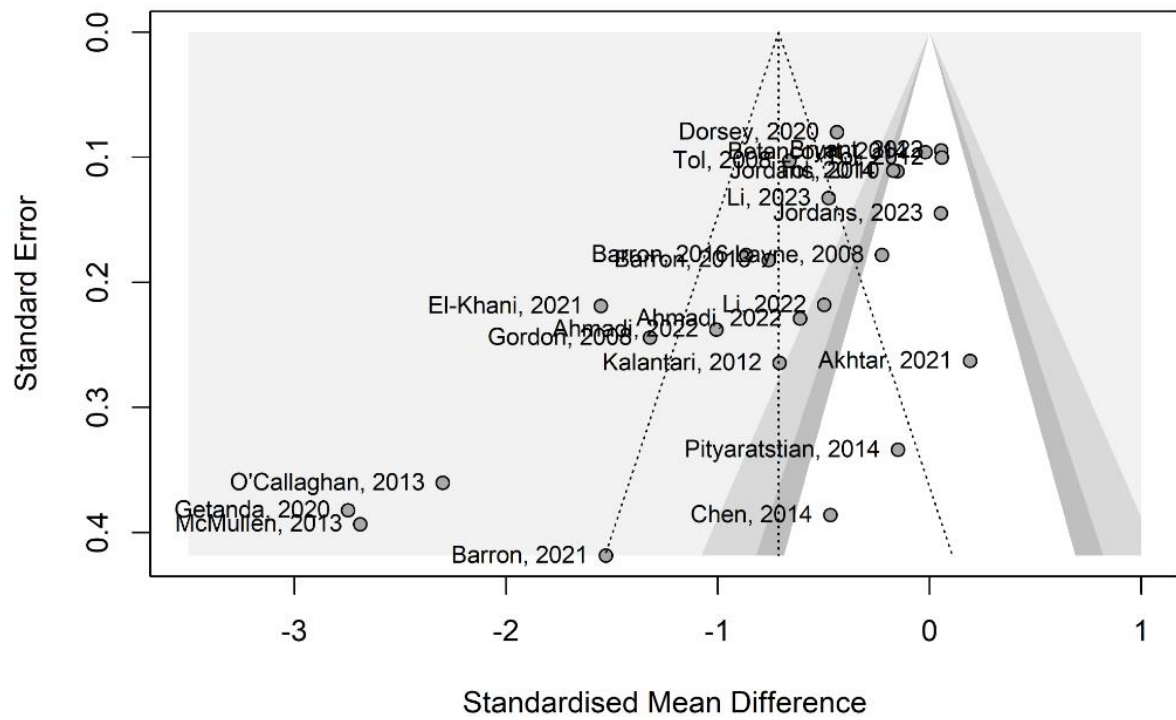

Supplement: S1 File — (PDF) [file pmen.0000533.s005.pdf]
