# Supplementary material for: Group psychosocial interventions for anxiety, depression, and post-traumatic stress disorder in children and adolescents in low- and middle-income countries: A realist systematic review and meta-analysis of randomised controlled trials
Source: PLOS Ment Health. 2026 Jan 29;3(1):e0000533. doi: 10.1371/journal.pmen.0000533 (PMC12854475; doi:10.1371/journal.pmen.0000533)

S2 File. Sensitivity analyses.

Sensitivity analyses – depression

Outlier studies:
Barron, 2016
Bryant, 2022
Jordans, 2023
Kaesornsamut, 2012
Layne, 2008
O'Callaghan, 2013
Tol, 2014
Zafar, 2015

Results with outliers removed:

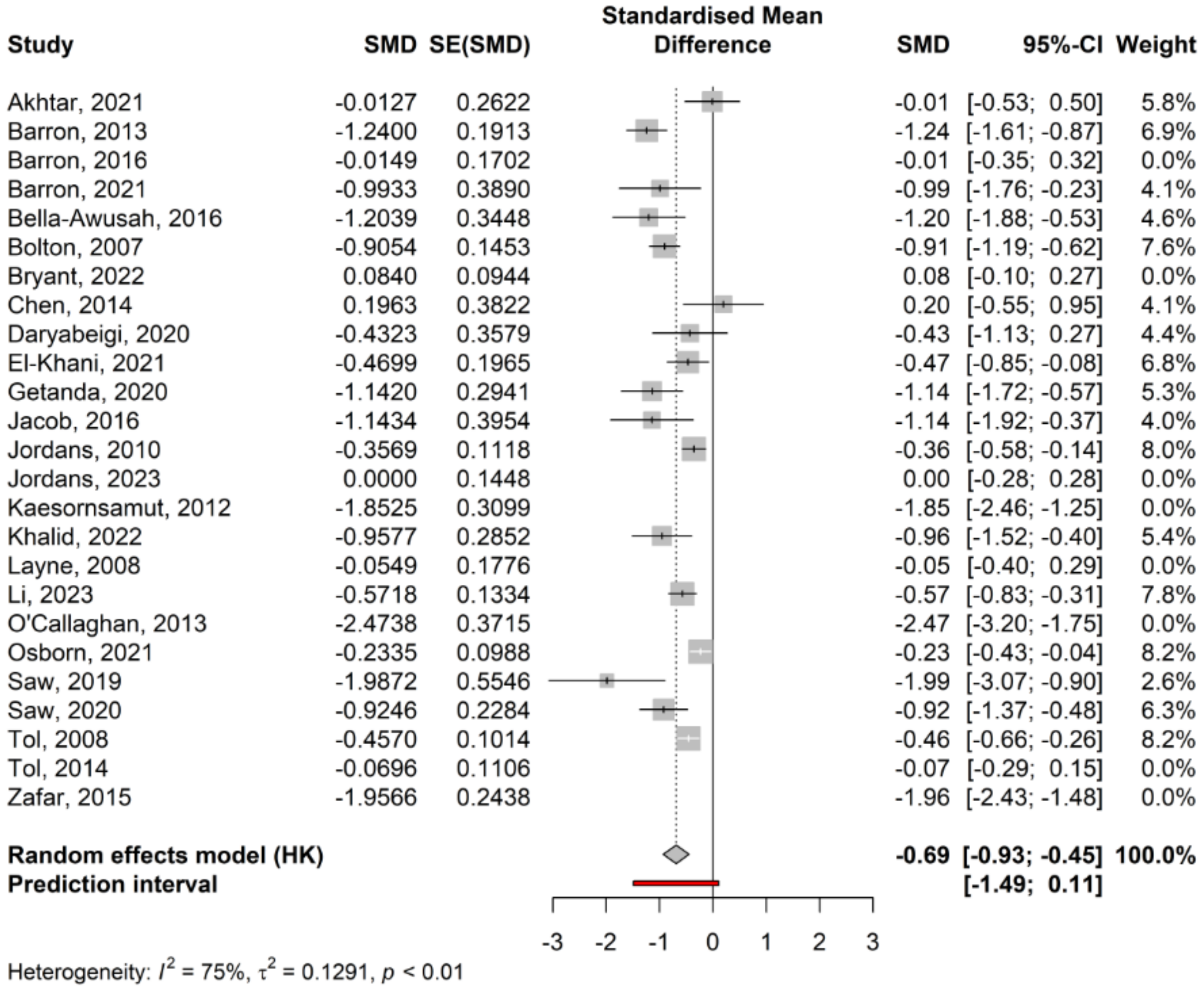

Influential studies (Baujat plot):

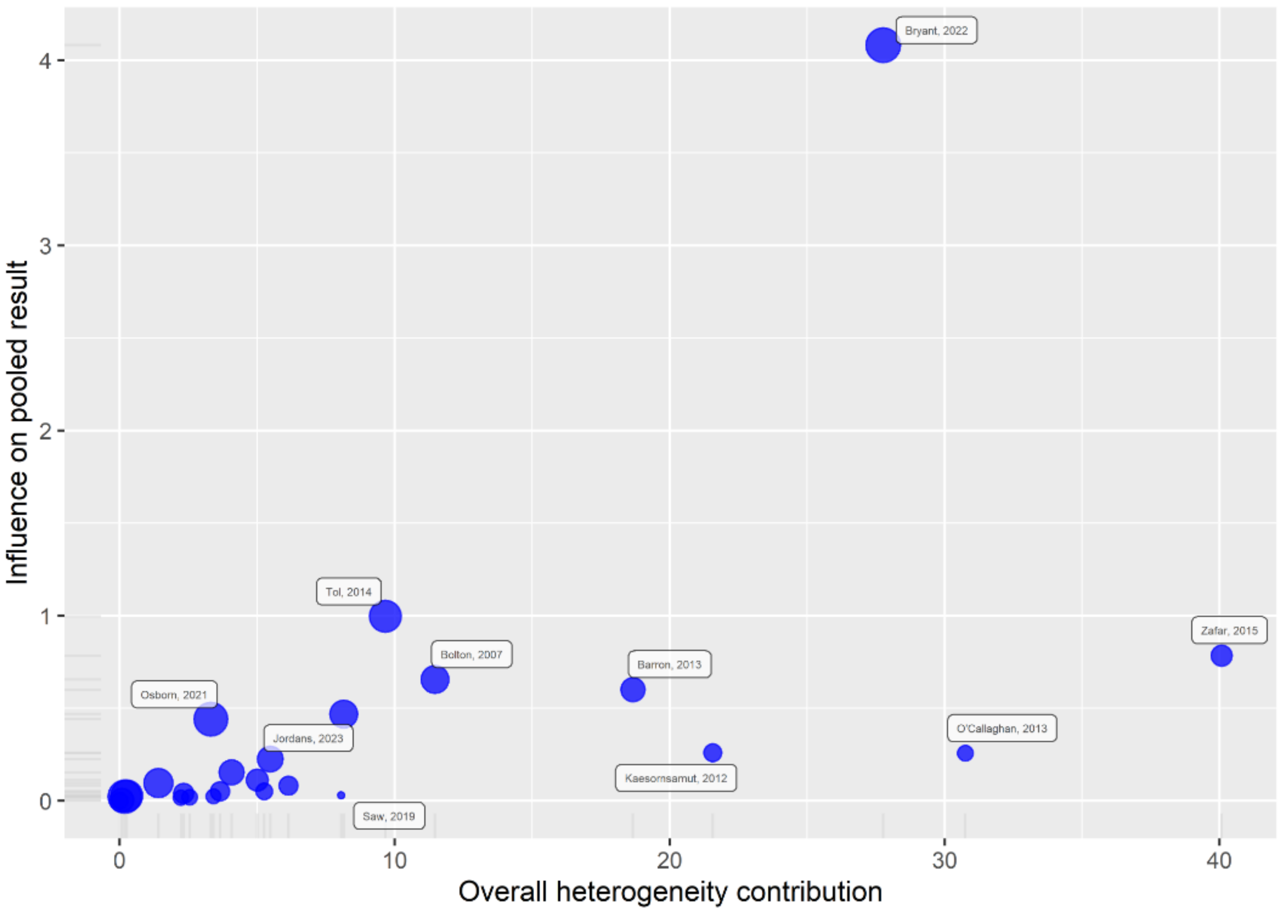

Sensitivity analyses – anxiety

Outlier studies:
Jordans, 2010
Peter, 2022

Results with outliers removed:

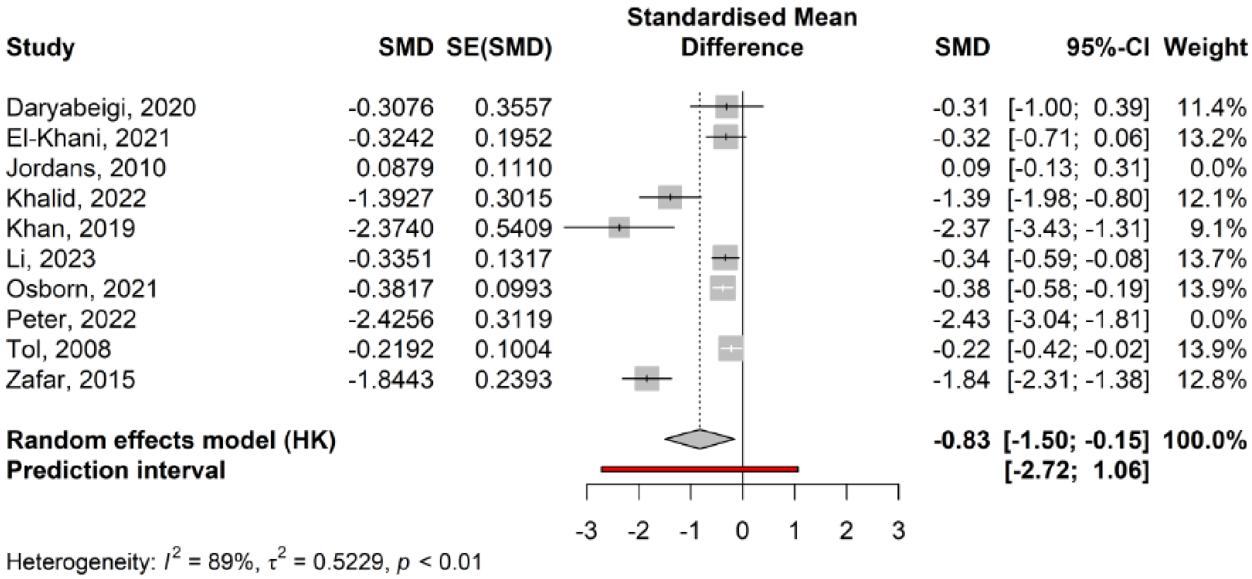

Influential studies (Baujat plot):

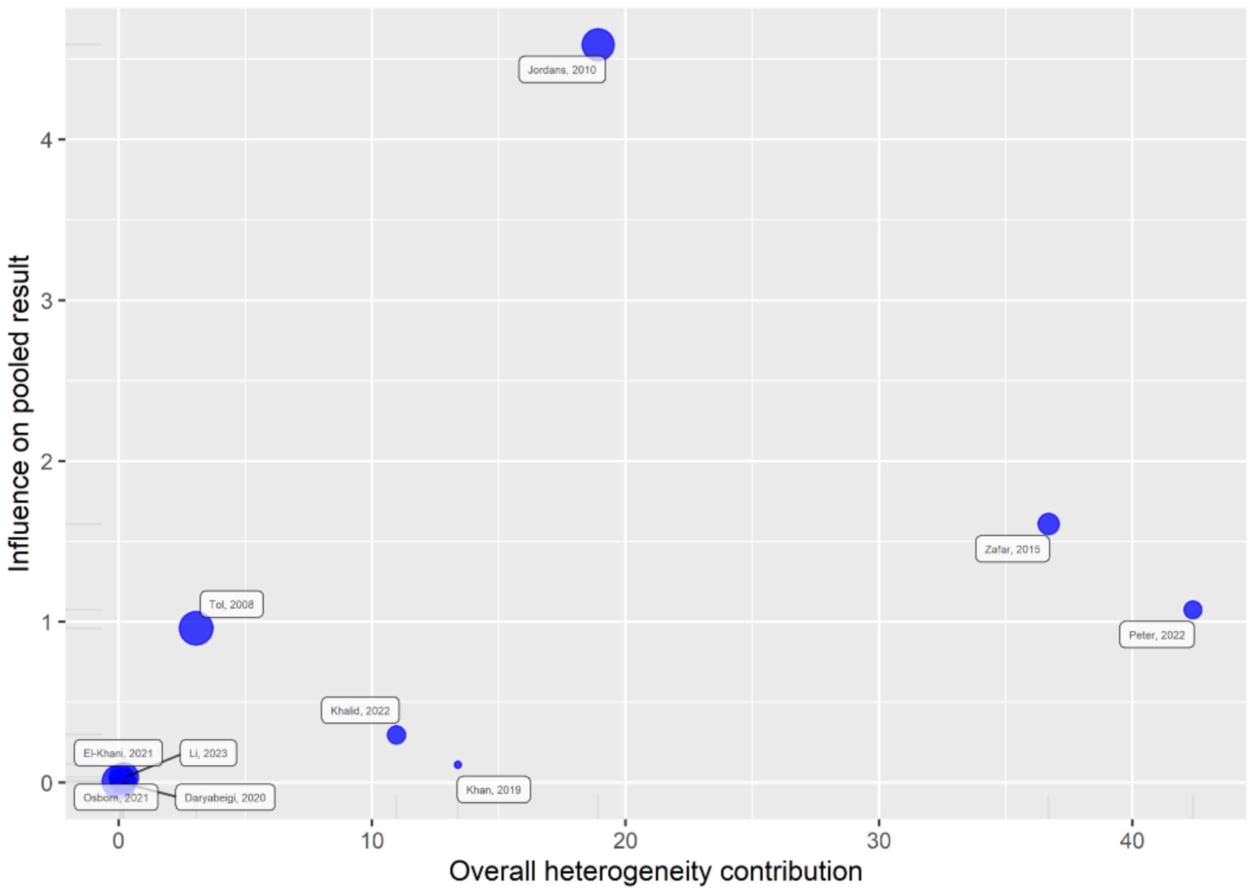

Sensitivity analyses - PTSD

Outlier studies:
Akhtar, 2021
Betancourt, 2014
Bryant, 2022
El-Khani, 2021
Getanda, 2020
Jordans, 2023
McMullen, 2013
O'Callaghan, 2013
Tol, 2012

Results with outliers removed:

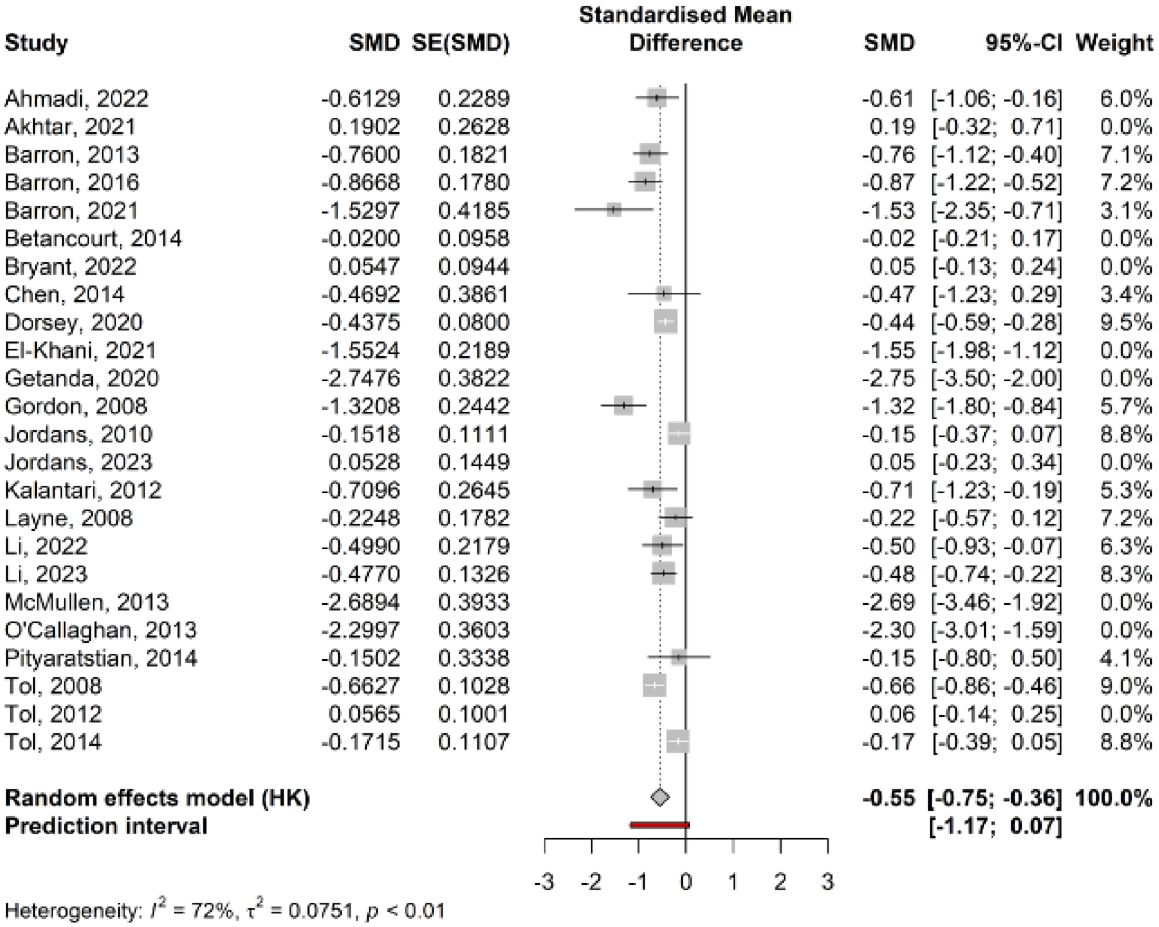

Influential studies (Baujat plot)

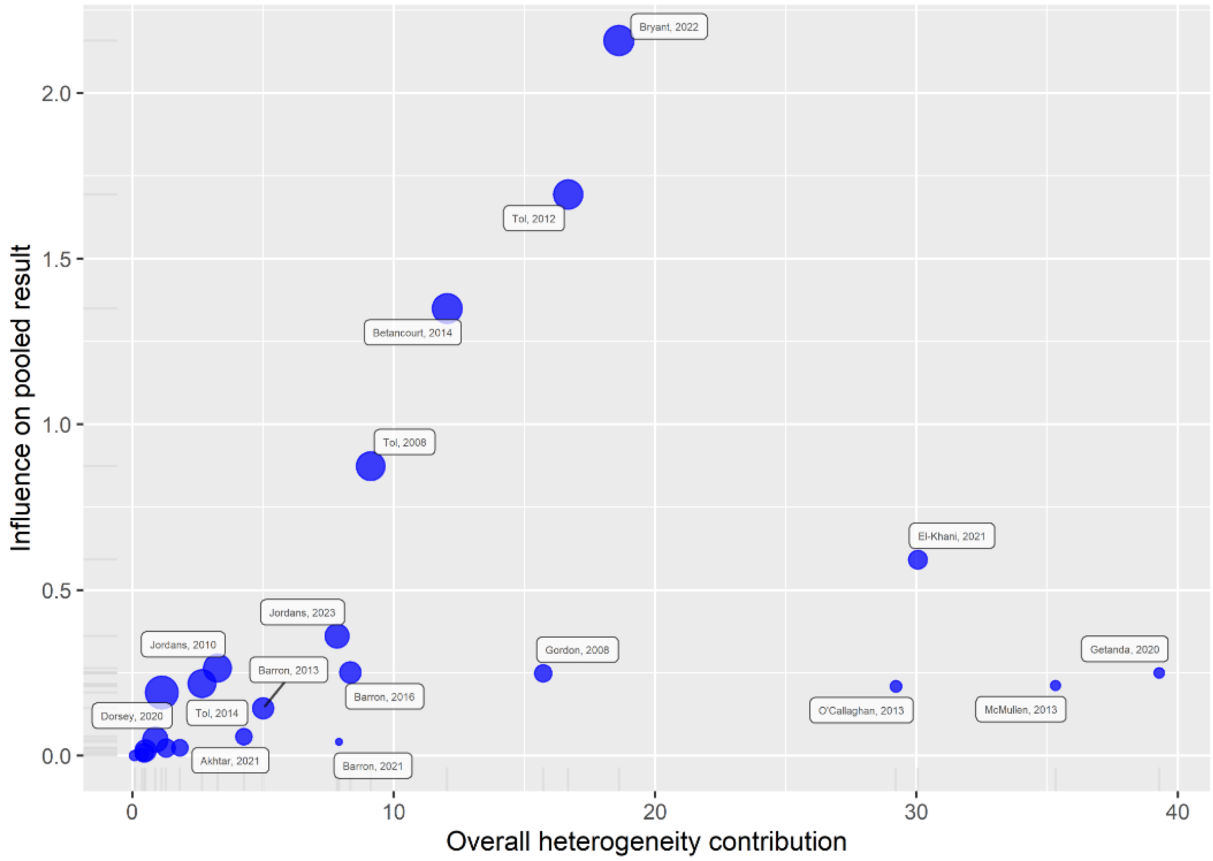

Supplement: S2 File — (PDF) [file pmen.0000533.s006.pdf]
