# Supplementary material for: Group psychosocial interventions for anxiety, depression, and post-traumatic stress disorder in children and adolescents in low- and middle-income countries: A realist systematic review and meta-analysis of randomised controlled trials
Source: PLOS Ment Health. 2026 Jan 29;3(1):e0000533. doi: 10.1371/journal.pmen.0000533 (PMC12854475; doi:10.1371/journal.pmen.0000533)
Supplement: S3 File — (PDF) [file pmen.0000533.s007.pdf]

S3 File. Meta-regressions.

Meta-regressions – depression

Variable	Chi-squared	df1	df2	p value
Treatment	3.50	2	22	0.048
Age group	5.23	1	23	0.032
Proportion Female	2.14	1	21	0.158
Conflict	5.13	1	23	0.033
Setting	1.50	2	22	0.246
Risk of Bias	1.42	2	22	0.262

Meta-regressions – anxiety

Variable	Chi-squared	df1	df2	p value
Age group	0.38	1	8	0.553
Proportion Female	0.16	1	7	0.705
Conflict	3.90	1	8	0.084
Risk of Bias	3.12	1	8	0.115

Meta-regressions - PTSD

Variable	Chi-squared	df1	df2	p value
Treatment	6.47	2	21	0.006
Age group	3.25	2	22	0.058
Proportion Female	0.12	1	22	0.732
Conflict	0.00	1	23	0.956
Setting	0.73	2	22	0.494
Risk of Bias	0.90	2	22	0.422
