# Supplementary material for: Group psychosocial interventions for anxiety, depression, and post-traumatic stress disorder in children and adolescents in low- and middle-income countries: A realist systematic review and meta-analysis of randomised controlled trials
Source: PLOS Ment Health. 2026 Jan 29;3(1):e0000533. doi: 10.1371/journal.pmen.0000533 (PMC12854475; doi:10.1371/journal.pmen.0000533)
Supplement: Attachment — Submitted filename: reviewer_comments.docx [file pmen.0000533.s010.docx]

We thank the reviewers for their thoughtful and helpful comments. We have revised the manuscript in response, and feel that the reviewer suggestions have helped to significantly improve the clarity and quality of the manuscript. Responses to individual suggestions are detailed below.

*1. The inclusion of studies spanning early childhood to late adolescence introduces developmental variability that likely influences intervention responsiveness. Consider explicitly categorizing age groups (e.g., early childhood, early adolescence, late adolescence) in the meta-regression analyses. The finding that older adolescents experienced greater reductions in depression may reflect their greater capacity for metacognitive engagement, whereas younger children may respond better to behaviorally oriented approaches. A brief developmental framing would enhance the interpretation of differential effects.*

Thank you, age groups have been categorised into early, middle, and late adolescence and this is reflected in the table of included studies. We have clarified this in the methods (226) and added a developmental framing in the discussion (451-454).

*2. The analysis does not account for gender-diverse, an increasingly important demographic in global mental health. While data may be limited, acknowledging this gap explicitly (perhaps in the limitations) would signal inclusiveness and transparency.*

Thank you, as none of our studies included any data for gender diverse participants, we have added this as an acknowledgement in the discussion (468-471).

*3. The manuscript notes the role of cultural adaptation in reducing stigma but would benefit from concrete illustrations or case examples (e.g., adaptations to local language, idioms of distress, or community delivery formats). This would strengthen the explanatory power of relevant CMOCs.*

Thank you, case examples from included studies are given in table 3 (final programme theories) where the CMOCs are illustrated in more detail.

*4. Given the realist aims, it is somewhat surprising that qualitative studies and grey literature were excluded. A brief rationale would clarify this decision and address potential reader concerns about omitted evidence.*

Thank you, we have now addressed this in the methods (142-144).

*5. Only 19 of the 38 included RCTs were deemed low risk of bias. Consider discussing the implications of this in more detail, particularly regarding the robustness of pooled effect sizes. The fact that adjusted effect sizes became non-significant after accounting for bias further underscores the need for cautious interpretation.*

Thank you, we have now addressed this in the limitations. Although adjusting for overall risk of bias did not significantly affect effect sizes, we have acknowledged that effect sizes did become non-significant after adjusting for small studies in the limit meta-analysis (524-530).

*6. A few covariates yielded non-significant results. These null findings could be contextualized further. For example, were these variables theoretically expected to influence outcomes? A more detailed discussion might help clarify their role or lack thereof.*

Thank you, we have commented further on examples of these in the results including school type (365-369).

*7. Where intervention effects were non-significant, consider hypothesizing potential reasons, such as ongoing trauma exposure, contextual stressors, or inadequate intervention intensity. This might also apply to the finding that CBT outperformed other modalities in some outcomes, despite sparse comparative trials.*

Thank you, we have clarified the wording to make this clearer in the results (330-332) and discussion (482-487).

*8. Since analyses are conducted at the study level, individual-level mechanisms cannot be inferred with confidence. For example, it is unclear whether older adolescents benefited more as individuals, or whether studies targeting them were better designed. Perhaps good to acknowledge the limitations of ecological fallacy and recommend future individual participant data meta-analyses to deepen this line of inquiry?*

Thank you, this was already mentioned in the limitations but we have reworded this section to aid clarity (534-538).

*9. The manuscript could briefly address the feasibility of implementing lay-delivered interventions in under-resourced school settings. Issues like training, supervision, and sustainability warrant mention, especially as this delivery model is central to many included studies.*

Thank you, we have now addressed this in the discussion (519-522).

*10. Consider acknowledging other potentially important moderators that were not assessed, such as facilitator training, session dose, frequency, and intervention duration and perhaps recommend them as areas for future investigation?*

Thank you, we have added this to the limitations (533-535).

*11. The covariate analysis partially advances the realist objectives by empirically exploring heterogeneity. However, linking the findings more explicitly back to the CMOCs and program theories in the discussion would improve narrative coherence. Highlighting how significant and null results map onto or challenge program theories would enrich interpretive clarity.*

Thank you, we have added more detail on this in the discussion, most relevant for the sections on age (454-455) and trauma exposure (488-492).
